# Molecular Hybridization as a Strategy for Developing Artemisinin-Derived Anticancer Candidates

**DOI:** 10.3390/pharmaceutics15092185

**Published:** 2023-08-23

**Authors:** Elena Marchesi, Daniela Perrone, Maria Luisa Navacchia

**Affiliations:** 1 Department of Environmental and Prevention Sciences, University of Ferrara, 44121 Ferrara, Italy; mrclne@unife.it; 2 Institute for Organic Synthesis and Photoreactivity (ISOF), National Research Council of Italy (CNR), 40129 Bologna, Italy

**Keywords:** molecular hybridization, artemisinin, dihydroartemisinin, artesunate, hybrids, click chemistry, anticancer activity

## Abstract

Artemisinin is a natural compound extracted from Artemisia species belonging to the Asteraceae family. Currently, artemisinin and its derivatives are considered among the most significant small-molecule antimalarial drugs. Artemisinin and its derivatives have also been shown to possess selective anticancer properties, however, there are several limitations and gaps in knowledge that retard their repurposing as effective anticancer agents. Hybridization resulting from a covalent combination of artemisinin with one or more active pharmacophores has emerged as a promising approach to overcome several issues. The variety of hybridization partners allows improvement in artemisinin activity by tuning the ability of conjugated artemisinin to interact with various molecule targets involved in multiple biological pathways. This review highlights the current scenario of artemisinin-derived hybrids with potential anticancer activity. The synthetic approaches to achieve the corresponding hybrids and the structure–activity relationships are discussed to facilitate further rational design of more effective candidates.

## 1. Introduction

**Figure 1:**
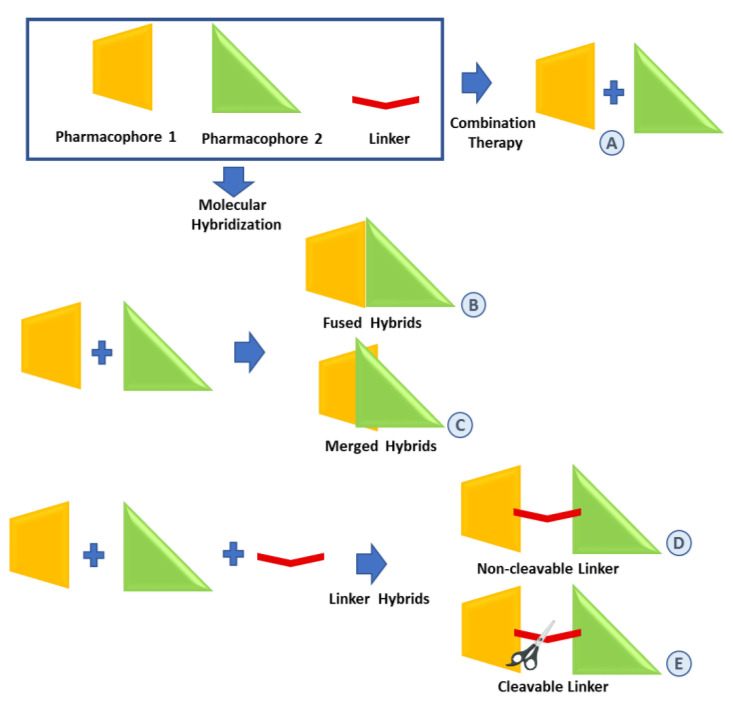
Strategies for the combination of pharmacophores. (**A**): combination therapy; (**B**–**E**): molecular hybridization approaches.

Cancer is considered to be one of the most life-threatening diseases throughout the world nowadays. World Health Organization estimated 10 million people worldwide to have died from cancer in 2020 [1]. Although chemotherapy is the conventional treatment for cancer, chemotherapeutic drugs frequently confer relevant levels of toxicity, are subject to drug resistance and lack bioavailability that limit their therapeutic potential and lower the rate of success in cancer [2,3,4,5]. To address these limitations, in recent decades significant efforts have been directed towards the development of combination therapies which involve the use of different drugs (Figure 1A) with diverse molecular mechanisms that can cooperatively affect and prevent cancer cells from developing drug resistance [6]. However, dose-limiting toxicities and drug–drug interactions can in turn limit the use of combination therapies. Hybrid molecules as multitarget anticancer agents have been envisaged as a valuable strategy to overcome the drawbacks of combination therapies as well as drug resistance [6,7].

Molecular hybridization is a modern strategy used in drug discovery for rational drug design [8]. Based on the covalent combination of pharmacophoric moieties of different bioactive substances, molecular hybridization produces new hybrid compounds that can be more than the simple sum of the parent pharmacophores [7,8]. In general, the concept of hybridizing various organic molecular motifs with different pharmacological activities can result in compounds with modified selectivity profile, different and/or dual modes of action, reduced undesired side effects, ability to overcome multidrug resistance and improved safety profile [9].

Typically, hybrid molecules containing two or more pharmacophoric units can be classified depending on the manner in which they are covalently linked. In the case of directly linked hybrids (fused hybrids in Figure 1B), the two molecular units are fused without using any linkers, that is, each molecule provides a functional group to build a link normally resulting in an enzymatically hydrolyzable ester, carbamate or amide. Merged hybrids (Figure 1C) result from the overlapping of structural motifs of both pharmacophores which may or may not retain the original functional activities. Linker hybrids are characterized by the presence of well-identifiable linkers classified as non-cleavable and cleavable. A non-cleavable linker (Figure 1D) leads to a hybrid that can be able to retain the biological activity and the affinity for the biological target of the different units by maintaining its structure or even convey a novel biological action. A cleavable linker (Figure 1E) is designed to release the parent agents with independent action under physiological or enzymatic conditions.

Over recent decades, molecular hybridization has been extensively used for the preparation of novel drug candidates with improved biological properties [9]. The relevance of a molecular hybridization strategy in cancer chemotherapy is evident by the number of hybrid compounds approved or under clinical trials [10,11].

Natural plant extracts represent a very important source of bioactive compounds for pharmacotherapy, especially for cancer and infectious diseases [12,13]. From the chemical point of view, numerous bioactive natural products from plants, such as taxoides deriving from the Pacific yew tree [14,15,16,17,18,19], are very interesting, due to the presence of reactive centers that enable the formation of covalent bonds with other molecules to give hybrid drugs. However, developing bioactive natural products into drugs has remained challenging, therefore, the use of natural compounds in the preparation of novel hybrid molecules has been considered an interesting strategy to empower their biological properties or to expand their biological space [20].

**Figure 2:**
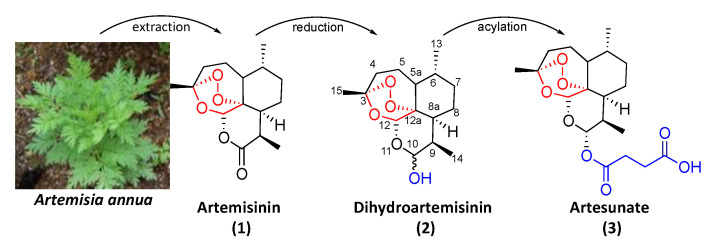
Picture of *Artemisia annua* and molecular structures of artemisinin (**1**), dihydroartemisinin (**2**) and artesunate (**3**).

*Artemisia annua* (Figure 2), a plant belonging to the Asteraceae family, has been safely used in traditional Chinese medicine over the centuries to treat a variety of fevers and respiratory tract infections. Artemisinin (1) (Figure 2), the bioactive component of *Artemisia annua*, and its semisynthetic derivatives, collectively called artemisinins, are very interesting compounds best known as antimalarial agents.

Dr. Youyou Tu’s research team discovered the effectiveness of *Artemisia annua* extracts in the inhibition of the parasite *Plasmodium falciparum* in the early 1970s [21]. In the next decades, artemisinin (**1**) and its derivatives were developed into drugs and became the gold standard for malaria treatment. More recent studies showed that artemisinins also exhibit interesting anticancer activities in vitro and in vivo against a wide range of cancer types [22,23,24,25,26], for instance, breast [27,28], ovarian [29], lung [30] and prostate [31,32].

Although the anticancer mechanism of artemisinin has not yet been fully elucidated [33,34], it has been reported that artemisinin exerts anticancer activity in the micromolar range mainly through apoptosis involving multiple targets and affecting multiple signaling pathways [23,25,26,35,36]. Non-apoptotic cell death mechanisms involved in anticancer activity of artemisinin, including oncosis-like cell death, autophagy and ferroptosis, have also been disclosed [34,35].

Some artemisinin derivatives have been identified as having potential for repurposing in anticancer therapy [23,26], [although several unfavorable properties including poor stability and water solubility and limited bioavailability prevent a large pharmacological application of artemisinin and its derivatives. To overcome artemisinin’s short half-life, and the consequent need for multiple doses each day, the World Health Organization has recommended artemisinin-based combination therapies (ACTs) as first-line treatment for uncomplicated malaria, by combining an artemisinin derivative and a slow-acting antimalarial drug [35,36,37,38,39]. Many studies have also been successfully devoted to the combination of artemisinins with chemotherapeutics or phytochemicals for cancer treatment [25,26,35,40,41]. Indeed, the synergistic effect of dihydroartemisinin (2) (Figure 2) in cancer combination therapies underlies some clinical trials involving dihydroartemisinin as a sensitizer agent [42].

On the other hand, molecular hybridization of an artemisinin moiety with other anticancer pharmacophores could be a valuable strategy not only to overcome short half-life, poor solubility and limited bioavailability but to provide combination therapies in a single multifunctional agent with enhanced activity and selectivity and reduced side effects and drug resistance compared to conventional treatments [6,9]. Over recent decades, numerous artemisinin-derived hybrids have been reported and evaluated for anticancer activity.

Considering our experience in design and synthesis of molecular hybrids for pharmacological applications [19,43,44,45,46], we decided to present herein a timely overview of the latest advances in the field of artemisinin-based hybrids for cancer disease. In particular, this review focuses on the chemical structure and biological evaluation of the most promising hybrids of artemisinin semisynthetic derivatives such as dihydroartemisinin (2) and artesunate (3) as well as their derivatives and highlights the strategies and the synthetic approaches to achieve the corresponding hybrids.

## 2. Chemical Features of Artemisinin and Selected Artemisinin Derivatives

Artemisinin (**1**) is a sesquiterpene lactone characterized by an endoperoxide bridge, located in the 1,2,4-trioxane ring (Figure 2). The endoperoxide moiety of artemisinin (**1**) has been shown to be pharmacologically relevant for both antimalarial and anticancer activities [26,33,47]. However, the lack of the endoperoxide moiety does not completely suppress anticancer activity [48].

Dihydroartemisinin (**2**) (Figure 2), a semisynthetic derivative of artemisinin (**1**), preserves the 1,2,4-trioxane ring whereas the C-10 carbonyl function of the lactone ring is modified into a lactol group by reduction with mild hydride-reducing agents [49]. The presence of the hemiacetal moiety improves the water solubility with respect to the parent artemisinin (**1**) and offers the chance for further chemical modifications. Dihydroartemisinin (**2**) presents enhanced antimalarial activity with respect to artemisinin (**1**) [26,50].

Artesunate (**3**) (Figure 2) is a more hydrophilic derivative of artemisinin (**1**) obtained by acylation of dihydroartemisinin by treatment with succinic anhydride under basic conditions [51]. Artesunate (**3**) shows improved solubility, absorption and pharmacokinetics with respect to artemisinin (**1**) and it is hydrolyzed under physiological conditions within minutes to its active metabolite dihydroartemisinin (**2**) [52].

## 3. Artemisinin Hybrids Based on Natural and Synthetic Pharmacophores

In the following section, we report selected examples of artemisinin-based hybrids in which artemisinin derivatives have been conjugated with biologically active natural compounds such as bile acids, steroids, alkaloids, terpenes, flavonoids, coumarins and cinnamic acids. Hybrids of artemisinin derivatives with the synthetic anticancer tamoxifen and sulfasalazine drugs are also reported. Table 1 briefly summarizes the hybrids discussed below, underlining the improvement in biological activities achieved through the hybridization.

### 3.1. Artemisinin–Bile Acid Hybrids

Bile acids are endogenous natural compounds with pharmacologically relevant activities also known to exert growth inhibition of several cancer cell lines through, among others, apoptosis, membrane alterations, modulation of nuclear receptors and oxidative stress [91]. However, the low cytotoxicity (IC_50_ > 100 μM) prevents their application as anticancer drugs and encourages their use in developing novel anticancer hybrids [91,92]. Indeed, bile acids present different available positions suitable for chemical modifications and are able to improve bioavailability [93], oral adsorption, cellular selectivity and site-specific delivery thanks to their amphiphilic properties and stability in dynamic pH variations, as well as the targeting of specific receptors [94,95].

**Scheme 1:**
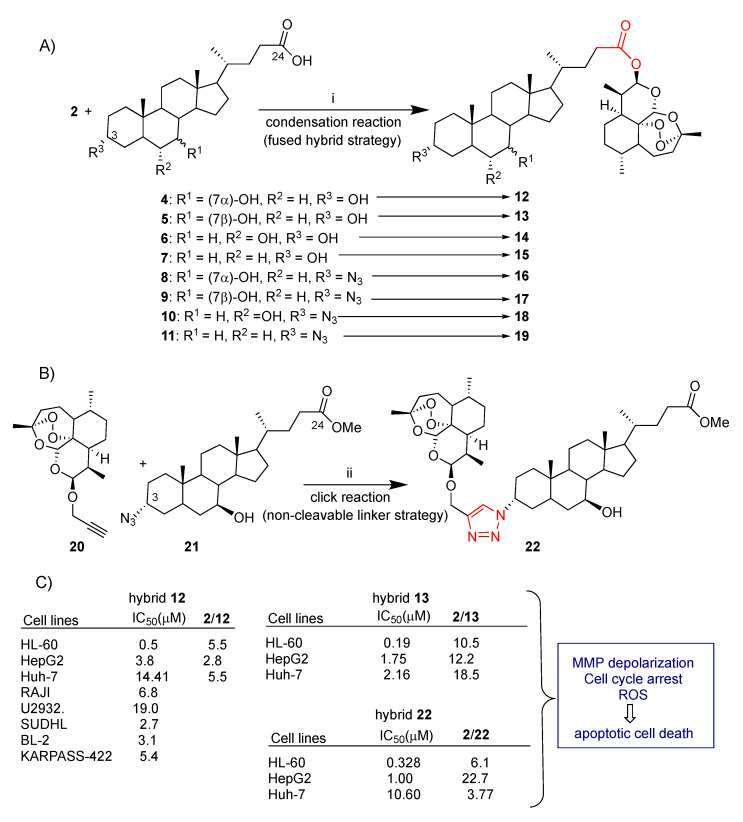
(**A**) Synthesis of dihydroartemisinin–bile acid hybrids **12**–**19** via condensation reaction: (i) EDC, DMAP, DMF, 25 °C, 18 h, 25–60% yield; (**B**) synthesis of dihydroartemisinin–bile acid hybrid **22** via click reaction: (ii) CuI 0.1 eq, r.t., 18 h, 28% yield; (**C**) selected biological data.

Recently, our research group explored convergent synthetic approaches to dihydroartemisinin–bile acid hybrids through both condensation reactions and click chemistry (Scheme 1A,B) [44]. Simple condensation reactions mediated by EDCI between dihydroartemisinin (**2**) and the appropriate bile acids **4**–**7** as well as 3-azido bile acid derivatives **8**–**11** revealed a successful synthetic strategy leading to the corresponding dihydroartemisinin–bile acid fused hybrids **12**–**19** in good yield. Hybrids **12**–**19** resulted from a direct conjugation of the dihydroartemisinin (**2**) lactol group with the C-24 carboxylic position of bile acids through a biologically labeled ester bond (Scheme 1A). Moreover, propargyl-dihydroartemisinin **20** was conjugated to 3α-azide derivative **21** via click chemistry to obtain **22** by the formation of a triazole linker which is stable under enzymatic and physiological conditions (Scheme 1B). Compound **20** was reacted with bile acid azide **21** in the presence of CuI to obtain the desired hybrid **22** in 28% yield. Though the click reaction is considered a high-yield one, in the present case a low yield was reported, probably due to the sensitivity of dihydroartemisinin (**2**) to the redox conditions employed in click chemistry. The linker can also play a role in the biological activity of the hybrids. In particular, the triazole ring is known to improve pharmacological, pharmacokinetic and physiochemical profiles of bioactive compounds, and it therefore can be somewhat considered a pharmacophore itself [96].

Dihydroartemisinin–bile acid hybrids **12**–**19** and **22** were tested for their in vitro cyctotoxicity against HL-60 leukemia cells. All hybrids showed enhanced cytotoxicity with respect to unconjugated dihydroartemisinin (**2**) with a dihydroartemisinin (**2**)/hybrid ratio ranging between 3 and 10.5 [44]. Further insights on mechanism were found for the most potent hybrid **13**, an ursodeoxycholic bile acid derivative, (dihydroartemisin (**2**)/**13** = 10.5) (Scheme 1C). Flow cytometry and Western blot analyses revealed that hybrid **13** induced apoptosis in HL-60 cells but no clear ROS production was detected in HL-60 cells treated with dihydroartemisinin (**2**) or hybrid **13** [44]. Chenodeoxycholic hybrid **12** was also tested in vitro against a selection of diffuse large B-cell lymphomas such as RAJI, U2932, SUDHL-4, KARPAS-422 and BL-2 cancer cell lines [53]. Hybrid **12** showed a significant concentration-dependent antiproliferative effect against all the diffuse large B-cell lymphomas tested (Scheme 1C). The mechanism investigation was limited to flow cytometry analysis with Annexin V-FITC staining that showed a highly predominant dose/time-dependent apoptotic mechanism of antiproliferative activity with a high early apoptosis percentage in SUDHL-4, KARPAS-422 and BL-2 cell lines [53].

Dihydroartemisinin (**2**) is known to significantly inhibit hepatocellular carcinoma cell growth in vitro and in vivo [97,98,99] but the poor stability limits its utilization. Therefore, dihydroartemisinin (**2**) conjugation with bile acids, also able to target hepatocyte transporters, may be desirable.

Almost all hybrids among **12**–**19** and **22** showed significant enhanced cytotoxic activity with respect to dihydroartemisinin (**2**) alone towards both HepG2 and Huh-7 liver cancer cell lines [44,45]. In particular, fused hybrid **13** was found to be 12.2- and 18.5-fold more potent than unconjugated dihydroartemisinin (**2**) in HepG2 and Huh-7 cells, respectively [44,45]. On the other hand, click hybrid **22**, linked at C-3 of ursodeoxycholic bile acid, showed higher selectivity towards HepG2 cells with respect to Huh-7 cells, being more potent than unconjugated dihydroartemisinin (**2**) by 22.7 and 3.77 times, respectively [44,45,46]. The improved stability in cell culture medium of hybrid **13** and **22** with respect to parent dihydroartemisinin (**2**), as a result of the conjugation, can in part account for the marked improved cytotoxicity. Indeed, HPLC-MS/MS studies demonstrated that both hybrids are completely stable in cell culture medium after 24 h whereas unconjugated dihydroartemisinin (**2**) decomposed up to 70% after 6 h [45,46]. A mechanism insight for hybrids **13** and **22** in hepatocellular carcinoma cells has been also reported [45,46]. Mechanism studies showed that hybrid **13**, similarly to dihydroartemisinin (**2**), induced G0/G1 arrest in both HepG2 and Huh-7 cells, elevated ROS levels in HepG2 but not in Huh-7 cells and caused depolarization of mitochondrial membrane potential (MMP) in both HepG2 and Huh-7 cells [45]. These effects may ultimately contribute to apoptosis induction. Similarly, click hybrid **22** induced G0/G1 arrest, ROS and mitochondrial membrane potential loss which may activate autophagy and in turn lead to apoptosis [46]. Thus, both hybrids may be potential drug candidates for hepatocellular carcinoma treatment.

**Scheme 2:**
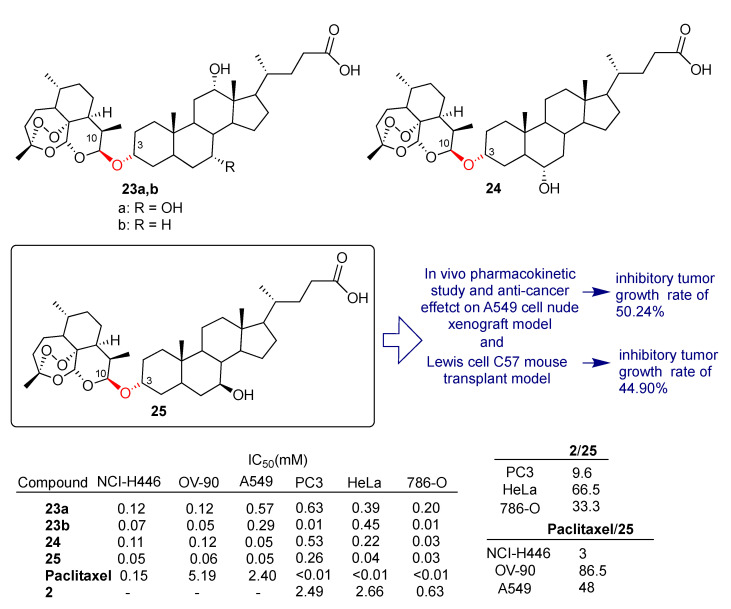
Structures of dihydroartemisinin–bile acid hybrids **23a**,**b**–**25** and selected biological data.

Recently, Zou et al. reported [54] the synthesis and the biological evaluation of a library of dihydroartemisinin–bile acid and dihydroartemisinin–steroid hybrids conjugated by condensation of dihydroartemisinin (**2**) and selected bile acids towards a broad panel of cancer cell lines including liquid and solid tumors. In particular, fused hybrids **23a**,**b**, **24** and **25** depicted in Scheme 2 were obtained by direct condensation mediated by BF_3_^.^Et_2_O of dihydroartemisinin (**2**) 10-OH with 3-OH of the appropriate bile acid. The condensation under the reaction conditions employed was found to be highly regio- and stereoselective. Hybrids **23a**,**b**, **24** and **25** displayed a marked enhanced anticancer activity compared to dihydroartemisinin (**2**) against PC3 (prostatic small cell carcinoma), HeLa (cervical cancer) and 786-O (renal cell carcinoma) cell lines and a higher cytotoxic activity with respect to reference drug paclitaxel in NCI-H446 and A549 (lung cancer) and OV-90 (ovarian cancer) cell lines (Scheme 2). In particular, fused hybrid **25**, obtained by conjugation of dihydroartemisinin (**2**) with ursodeoxycholic bile acid, was found to be the most active of the series, being 86.5- and 48-fold more potent than reference drug paclitaxel in OV-90 and A549 cell lines, respectively (Scheme 2). The pharmacokinetic profile of hybrid **25** was also obtained by using male Sprague Dawley rats. Hybrid **25** displayed acceptable maximum concentration and oral exposure. Moreover, a short half-life of 0.8 h, typically associated with artemisinin compounds, and good plasma clearance were also reported.

The A549 cell nude mouse xenograft model and Lewis cell C57 mouse transplant model were used to study the in vivo antitumor effect of hybrid **25**. The A549 cell xenograft model showed that hybrid **25** significantly reduced tumor growth, with an inhibitory rate of 50.24%. In the Lewis cell C57 mouse transplant model, a 44.90% inhibitory rate was observed at a dosage of 12 mg/kg, which was significantly higher than that of the paclitaxel group (14.91% at a dosage of 10 mg/kg). It was also demonstrated by flow cytometry analysis that hybrid **25** significantly increased the concentration of CD3 (*p* < 0.05) and decreased the concentration of CD19 (*p* < 0.01). A FOXP3 experiment showed that hybrid **25** significantly increased the concentration of Tregs (*p* < 0.01). The overall data indicated that the conjugation of dihydroartemisinin (**2**) with ursodeoxycholic bile acid may have some relevance in immunotherapy.

**Scheme 3:**
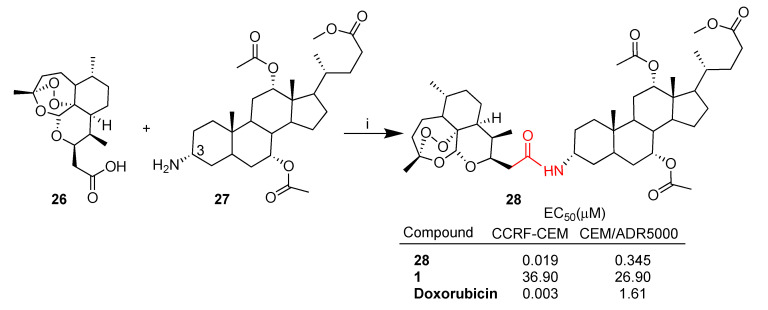
Synthesis of hybrid **28** and selected biological data. Reaction conditions: (i) EDC, DMAP, DCM, 24 h, 86% yield.

Letis et al. reported [55] the synthesis of a series of artemisinin–cholic bile acid hybrids through condensation reactions mediated by EDCI. The synthesis of the most active hybrid **28** prepared by conjugation of deoxoartemisinin **26** to 3α-NH_2_ cholic acid derivative **27** is depicted in Scheme 3. The newly prepared hybrids were tested towards sensitive CCRF-CEM and multidrug-resistant CEM/ADR5000 lymphoblastic leukemia cells. Improved cytotoxic activity compared to artemisinin (**1**) itself was found in all cases [55]. The combination of several semisynthetic dihydroartemisinin derivatives with different cholic bile acid derivatives allowed insights into the structure–activity relationships.

Compound **28** resulted the most potent hybrid of the series in both CCRF-CEM and CEM/ADR5000 cell lines with an IC_50_ value of 0.019 μM and 0.345 μM, respectively (Scheme 3). Hybrid **28** showed significant cytoselectivity towards sensitive CCRF-CEM, being 18-fold more potent than in multidrug-resistant CEM/ADR500 cancer cells. Moreover, hybrid **28** was found to be 4.6-fold more potent than reference drug doxorubicin (IC_50_ = 1.61 μM) and far more active than unconjugated artemisinin (**1**) (IC_50_ = 26.90 μM) in multidrug-resistant CEM/ADR5000 cancer cells (Scheme 3). The structure–activity relationship study also highlights some key points. Particularly, the 3α-orientation of the cholic acid skeleton and the incorporation of the artemisinin-derived carboxylic acid **26**, forming a two-carbon chain linker between the artemisinin and the cholic acid moieties, contribute to the enhancement of the cytotoxic activity of the amide hybrid **28**.

The chemical hybridization of drug compounds with bile acid skeletons appears very attractive since bile acids may improve the bioavailability of parent compounds [95]; nevertheless, so far, in vivo experiments are limited to dihydroartemisinin–ursodeoxycholic acid **25** reported by Zou et al. in 2021 [54].

### 3.2. Artemisinin–Quinoline and Quinazoline Hybrids

**Scheme 4:**
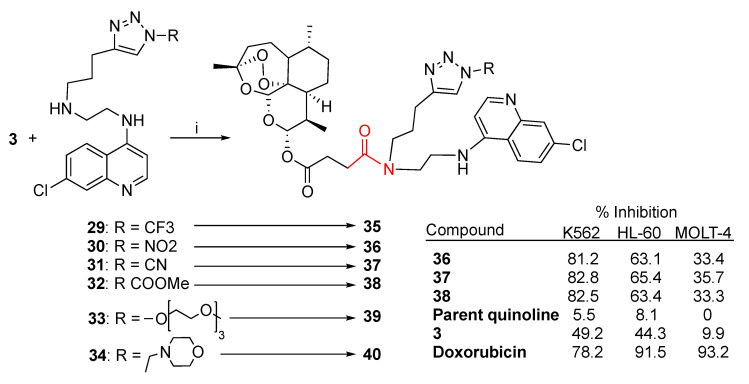
Synthesis of artesunate–quinoline hybrids and selected biological data. Reaction conditions: (i) EDC, DMAP, DCM, 0 °C to r.t., overnight, 33–69% yield.

Quinoline and quinazoline alkaloids are two important classes of *N*-heterocyclic compounds with a wide range of pharmaceutical properties including antibacterial, antiviral, anticancer and antiparasitic effects [100,101,102,103,104,105]. Integration of a quinoline moiety can improve physical and chemical properties as well as pharmacological behavior, therefore, the quinoline ring represents an attractive scaffold in drug design. Quinoline derivatives have been vastly explored as partners in artemisinin combination therapy. Recently, Hermann et al. reported [56] the synthesis of a small library of artesunate–quinoline hybrids obtained through condensation reactions mediated by EDCI and DMAP between artesunate (**3**) and the series of chloroquinoline triazole derivatives **29**–**34** (Scheme 4). The newly synthesized hybrids **35**–**40** were tested against several leukemia cell lines including K562, HL-60 and MOLT-4 (Scheme 4). The best improvement of % growth inhibition compared to the parent quinoline derivative and artesunate (**3**) was shown by hybrids **36**–**38**. Notably, hybrids **36**–**38** showed comparable anticancer activity towards K562 cells with respect to the antileukemia reference drug doxorubicin (Scheme 4).

It is worth noting that hybrids **36**–**38** also showed a marked inhibitory activity against SARS-CoV-2 with EC_50_ = 13–19 μM [56]. This result should encourage the design of further hybrids with combined anticancer and antiviral properties.

**Scheme 5:**
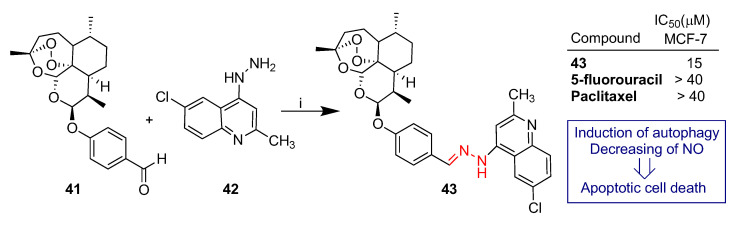
Synthesis of artemisinin–quinoline hybrids **43** and selected biological data. Reaction conditions: (i) MeOH, r.t., 7 h.

Yao et al. reported [59] the synthesis and biological evaluation of dihydroartemisinin–quinoline hybrid **43** (Scheme 5). Hybrid **43** was prepared by condensation of dihydroartemisinin–benzaldehyde derivative **41** and 4-quinolylhydrazine **42** and tested against the MCF-7 breast cancer cell line (Scheme 5). Hybrid **43** showed significantly higher anticancer activity than that of reference drugs 5-fluorouracil and paclitaxel with an IC_50_ ≈ 15 μM (Scheme 5). A mechanism insight on cell death revealed that hybrid **43** inactivates the c-Jun N-terminal kinase signaling pathway, leading to the induction of autophagy and apoptosis with NO generation restricted [60].

**Scheme 6:**
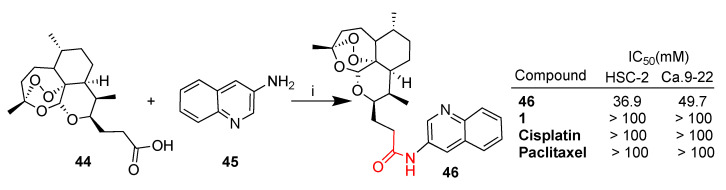
Synthesis of quinoline hybrid **46** and selected biological data. Reaction conditions: (i) EDC, DMAP, DMF, r.t., 3 h, 89% yield.

Deoxoartemisinin derivative **44** was conjugated with 3-aminoquinoline **45** through a condensation reaction (Scheme 6) [57]. The resulting hybrid **46** obtained in high yield was tested in vitro against several cancer cell lines including HSC-2 and Ca.9-22 oral squamous carcinoma cells [57]. Hybrid **46** showed significantly higher cytotoxic activity with respect to parent artemisinin (**1**) as well as to standard drugs such as cisplatin and paclitaxel (Scheme 6).

**Scheme 7:**
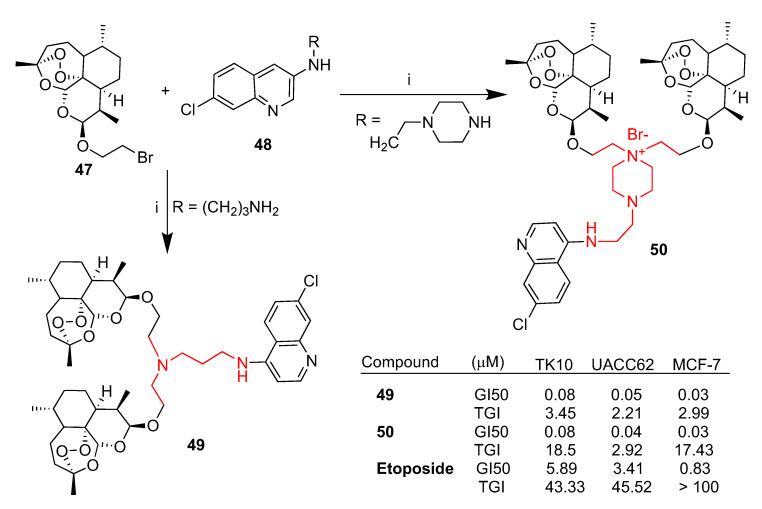
Synthesis of dihydroartemisinin–quinoline hybrids 49 and 50 and selected biological data. Reaction conditions (i): DMF, 90–110 °C, 6–8 h.

Quinoline–bis(dihydroartemisinin) hybrids 49 and 50 [61] were prepared as shown in Scheme 7 and tested in vitro against TK10 (renal), UACC62 (melanoma) and MCF7 (breast) cancer cell lines [62]. The cytotoxic activity was expressed as 50% growth inhibition (GI_50_) and drug concentration resulting in total growth inhibition (TGI). The anticancer agent etoposide was used as a reference standard drug. Both quinoline–bis(dihydroartemisinin) hybrids 49 and 50 showed anticancer activity against all cancer cell lines tested with GI_50_ values in the range of 0.03–0.08 μM and TGI values in the range of 2.21–18.50 μM. Both hybrids were found to be much more potent than the reference drug etoposide (GI_50_ = 0.83 and 5.89 μM, respectively; TGI = 43.33 and >100 μM, respectively) (Scheme 7). Pharmacokinetic studies were also performed in order to support further desirable in vivo studies. Maximum concentrations of 279 ng/mL and 210 ng/mL in tumors were reached within 16.7 and 20.0 min for hybrids 49 and 50, respectively, and both hybrids showed a faster blood clearance compared to dihydroartemisinin (2) (3.06 and 4.78 L/min/kg vs. 1.19 L/min/kg for 2). The overall data indicated that both hybrids 49 and 50 can be considered as lead compounds for further in vivo investigations.

The synthesis of a small library of artesunate–indoloquinoline hybrids prepared by a condensation reaction between artesunate (**3**) and the appropriate indoloquinoline derivative in the presence of EDCI and 1-hydroxybenzotriazole was reported [58].

**Scheme 8:**
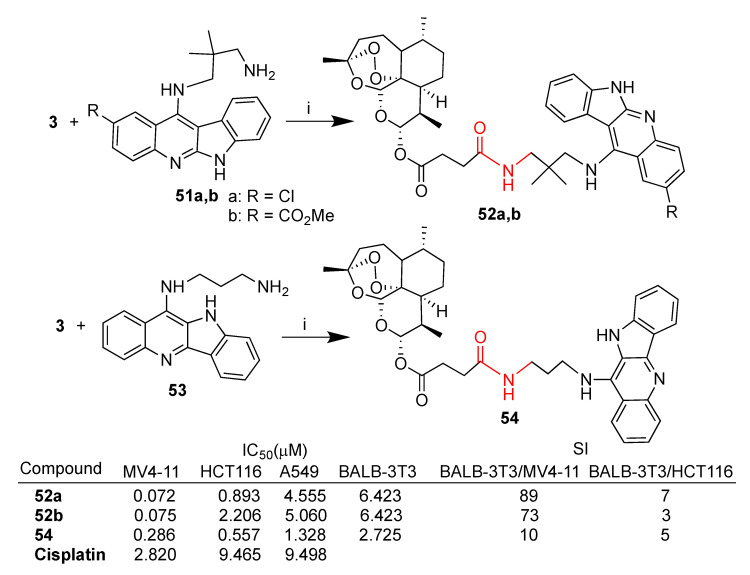
Synthesis of artesunate–indoloquinoline hybrids **52a**,**b** and **54** and selected biological data. Reaction conditions: (i) DCM, EDC, OHBt, r.t., 6 h, 40–50% yield.

All hybrids were tested in vitro against a selection of cancer cell lines such as leukemia (MV4-11), lung (A549) and colon cancer (HCT-116) as well as healthy cells (BALB-3T3) [58]. The synthesis of selected hybrids **52a**,**b** and **54** is reported in Scheme 8. Hybrids **52a**,**b** and **54** exhibited promising activity against MV4-11 and HCT-116, being significantly more potent than the reference drug cisplatin and showing a good selectivity index with respect to healthy cells (BALB-3T3) (Scheme 8). In the case of the A549 cancer cell line, the hybrids were still more potent than cisplatin but less selective, having IC_50_ comparable to that reported for the healthy cell line BALB-3T3. Hybrids **52a**,**b** showed the best activity and selectivity profile against leukemia MV4-11 cells with IC_50_ of 0.072 and 0.075 μM and selectivity index, with respect to the healthy cell line BALB-3T3, of 89 and 73, respectively (Scheme 8).

**Scheme 9:**
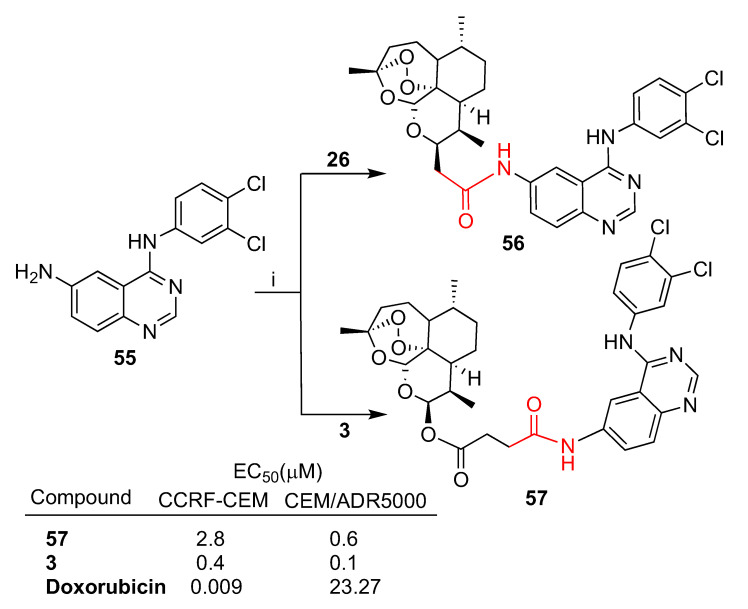
Synthesis of artemisinin–quinazoline hybrids **56** and **57** and selected biological data. Reaction conditions: (i) DMF, EDC, DIPEA, OHBt, r.t., 1–3 days, 68–64% yield.

Fröhlich et al. reported [63] the synthesis of hybrids **56** and **57** by conjugation of quinazoline **55** with deoxoartemisinin **26** and artesunate (**3**), respectively (Scheme 9). Both hybrids were tested for their in vitro cytotoxic activity towards sensitive wild-type CCRF-CEM and multidrug-resistant P-glycoprotein-overexpressing CEM/ADR5000 leukemia cells. Deoxoartemisinin–quinazoline hybrid **56** showed low antileukemia activity towards both cell lines tested. In contrast, artesunate–quinazoline hybrid **57** exhibited an antileukemia effect with EC_50_ values of 2.8 μM in CCRF-CEM and 0.6 μM in CEM/ADR5000, similar to that of parent artesunate (**3**). Hybrid **57**’s activity was found to be 45 times higher than that of reference drug doxorubicin (EC_50_ = 23.27 μM) in multidrug-resistant CEM/ADR5000 cells (Scheme 9).

**Scheme 10:**
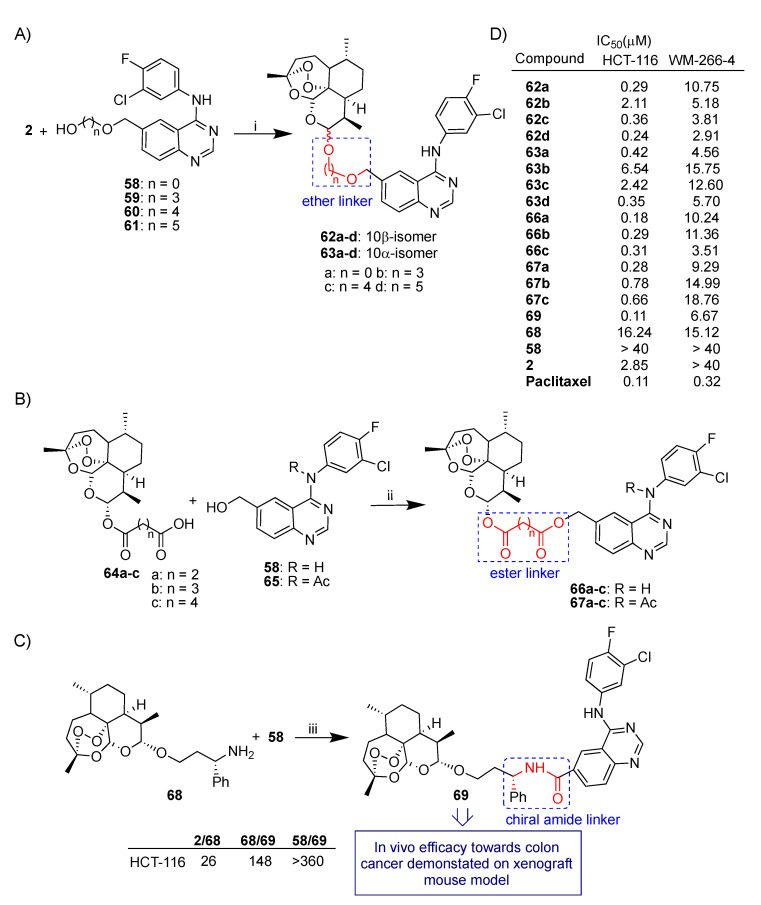
(**A**–**C**): synthesis of artemisinin–quinazoline hybrids; (**D**) selected biological data. Reaction conditions: (i) BF_3_^.^Et_2_O, DCM, EDC, r.t., overnight, 19–33% yield; (ii) Et_3_N, DCM, EDC, r.t., 63–77% yield; (iii) EDC, DMAP, DPEA, DCM, 40 °C, overnight, 42% yield.

Wang et al. recently reported a wide study on the synthesis and anticancer activity of several series of artemisinin–quinazoline hybrids [64] featuring ether (Scheme 10A) or ester (Scheme 10B) flexible linkers of different lengths besides a more rigid amide chiral linker (Scheme 10C). As depicted in Scheme 10A, dihydroartemisinin (**2**) was conjugated through condensation reactions with quinazoline derivatives **58**–**61** to obtain 10β-anomer hybrids **62a**–**d** and 10α-anomer hybrids **63a**–**d.** Hybrids **62a**–**d** and **63a**–**d** were tested in vitro against two cancer cell lines, HCT-116 (colon) and WM-266-4 (melanoma). Interestingly, in most cases 10β-anomer hybrids **62a**–**d** showed higher antiproliferative activity compared to 10α-anomers hybrids **63a**–**d** in both cancer cell lines tested, indicating a possible role of the stereochemistry of dihydroartemisinin (**2**) C-10 anomeric carbon in the biological activity. On the contrary, a clear correlation between the linker length and the bioactivity could not be assessed. The 10β-anomer **62d** with the longest linker alkyl chain was the most active hybrid, being ca. 12- and >14-fold more potent than parent dihydroartemisinin (**2**) towards HCT-116 and WM-266-4 cancer cell lines, respectively (Scheme 10D). Condensation reactions of artesunate homologues **64a**–**c** with quinazoline **58** as well as its corresponding *N*-acetylated quinazoline **65** led to hybrids **66a**–**c** and **67a**–**c**, respectively (Scheme 10B). A slight influence of the length of the linkers on the bioactivity can be observed (Scheme 10D). Moreover, *N*-hydrogen hybrids **66a**–**c** were found to be more potent than the *N*-acetylated homologues **67a**–**c** towards both cancer cell lines tested.

A condensation reaction of amino dihydroartemisinin derivative **68** with quinazoline **58** led to hybrid **69** characterized by an (*S*)-alkylamide linker (Scheme 10C) [64]. Hybrid **69** showed the best cytotoxic activity towards the HCT-116 colon cancer cell line of all hybrids tested with IC_50_ = 0.11 μM, comparable to that of reference drug paclitaxel (Scheme 10D) [64].

Notably, molecular hybridization was particularly efficient in enhancing the cytotoxic activity in HCT-116. Thus, hybrid **69** was 148-fold more potent than parent dihydroartemisinin derivative **68** (and in turn 26-fold more potent than dihydroartemisinin (**2**) itself) as well as > 360-fold more potent than unconjugated quinazoline **58** (Scheme 10D).

Hybrid **69** was further studied for in vivo anticolon cancer activity. A study on xenograft mice disclosed that hybrid **69** actually delayed tumor growth after 18 days of treatment, without affecting the weight of mice compared with the control group [64]. These results highlight hybrid **69** as a very interesting structural lead compound for the development of new anticancer drugs.

### 3.3. Artemisinin–Nitrogen Mustard Hybrid

Nitrogen mustard compounds are DNA-alkylating agents characterized by antiproliferative potency. Even though several types of nitrogen mustard derivatives such as chlorambucil, melphalan and cyclophosphamide are used in clinical applications, their non-specific DNA alkylation as well as the high dose required to reach effective plasma concentrations increase the risk of drug toxicity and drug resistance [106]. Therefore, the conjugation of nitrogen mustards with bioactive natural molecules characterized by low toxicity can lead to improved antitumor effect and selectivity and reduced toxicity. In recent decades, many nitrogen mustard hybrids have been reported and nitrogen mustard molecular hybridization has turned out to be an effective strategy to obtain anticancer agents with increased activity, reduced toxicity and improved physicochemical properties [106].

Recently, Dai et al. reported [65] a novel class of nitrogen mustard hybrids obtained by conjugation with artemisinin derivatives.

**Scheme 11:**
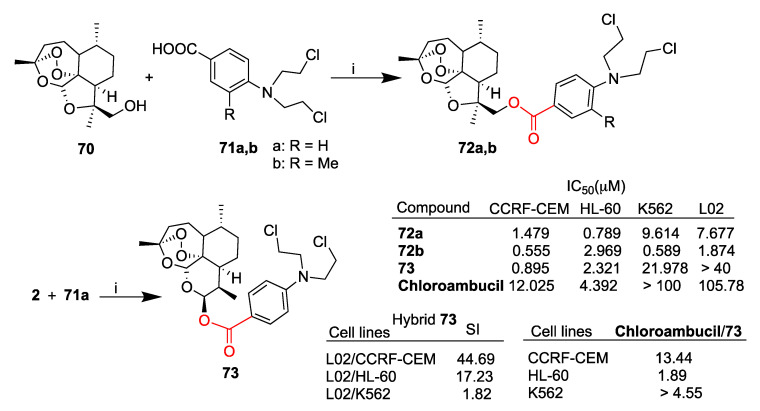
Synthesis of artemisinin–nitrogen mustard hybrids and selected biological data. Reaction conditions: (i) DMAP, EDC, DCM, r.t., overnight, 30–60% yield.

In particular, the ring contracted artemisinin derivative **70** and dihydroartemisinin (**2**) were conjugated with a series of nitrogen mustards, among other compounds **71a**,**b**, through condensation reactions mediated by DMAP and EDCI (Scheme 11). All novel hybrids were tested in vitro against a selection of cancer cell lines including leukemia CCRF-CEM, HL-60 and K562 as well as healthy liver L02 cells [65]. Among others, hybrids **72a**,**b** and **73** depicted in Scheme 11 were found to be the most promising compounds, displaying significantly higher activity with respect to the reference drug chloroambucil and good selectivity compared to healthy liver L02 cells. In particular, dihydroartemisinin-derived hybrid **73** displayed the best selectivity towards CCRF-CEM cell lines, being ca. 14-fold more active than chloroambucil and ca. 44-fold less toxic than in L02 healthy cells. Further investigation on the cell death mechanism suggested that hybrid **73** promoted both early and late apoptosis. Moreover, Western blot assay revealed that hybrid **73** downregulated the expression of the antiapoptotic protein Bcl-2, while the expression of procaspase-3 and cleaved caspase-3 did not show any significant changes. Additionally, hybrid **73** significantly improved the activity of glutathione peroxidase to reduce total ROS levels, therefore inhibiting cell proliferation. The overall data highlight hybrid **73** as a lead compound for possible clinical applications.

**Scheme 12:**
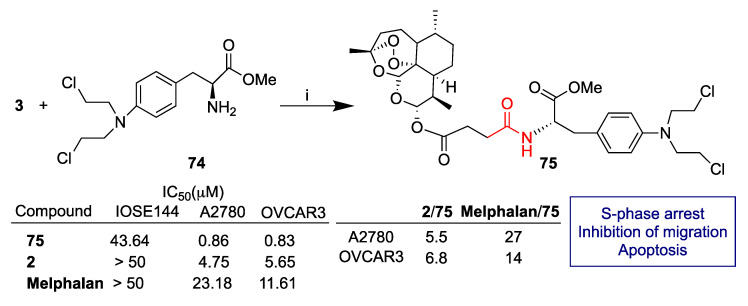
Synthesis of artesunate hybrid **75** and selected biological data. Reaction conditions: (i) DMAP, EDC, DCM, r.t., overnight, 88% yield.

Li et al. reported [66] the synthesis of artemisinin-derived hybrids obtained by conjugation of several chemotherapeutic alkylating agents including, among others, chloroambucil, melphalan and flutamide. In particular, molecular hybridization via the condensation reaction of artesunate (**3**) with melphalan methyl ester **74** led to melphalan-derived hybrid **75** that showed marked enhanced activity against A2780 and OVCAR3 ovarian cancer cell lines with respect to the unconjugated dihydroartemisinin (**2**) and melphalan drug (Scheme 12). Further mechanism studies showed that hybrid **75** led to S-phase arrest, apoptosis and inhibition of migration. Moreover, hybrid **75** was found to modulate the expression of proteins involved in cell cycle progression, apoptosis and epithelial–mesenchymal transition. In vivo studies in mice highlighted that hybrid **75** can inhibit growth, intraperitoneal dissemination and metastasis of ovarian cancer cells without observable toxic effects. Therefore, hybrid **75** can be considered a promising lead compound for the treatment of ovarian cancer.

### 3.4. Artemisinin–Tyrosol Hybrids

**Scheme 13:**
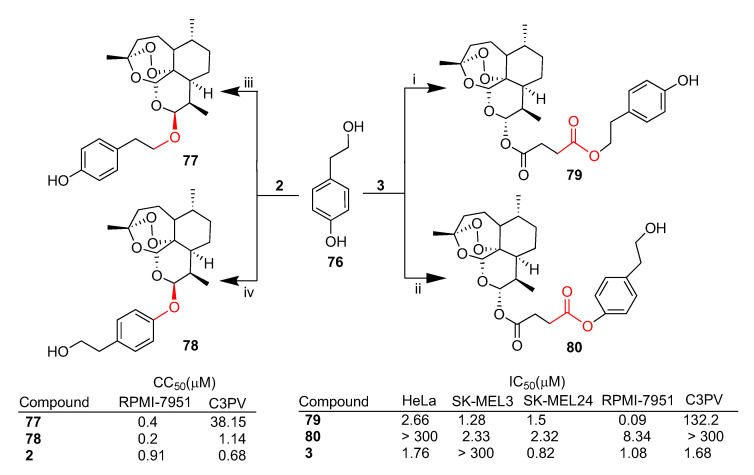
Synthesis of artemisinin–tyrosol hybrids and selected biological data. Reaction conditions: (i) K_2_CO_3_, DMF, 90 °C, overnight, 50% yield; (ii) DMAP, DCC, DCM, r.t., overnight, 53% yield; (iii) Et_2_O, BF_3_^.^Et_2_O, 0 °C, overnight, 48%; (iv) PPh_3_, DIAD, DMF, r.t., overnight, 60% yield.

Tyrosol (**76**, Scheme 13) is a simple phenolic component of olive oil with well-established antioxidant and anti-inflammatory properties. Although some evidence of antiproliferative activity has been reported in the literature, tyrosol **76** is mainly employed in non-malignant diseases such as diabetes and cardiovascular disorders [107].

Botta et al. recently reported [67,68] the synthesis of a series of artemisinin–tyrosol hybrids and their in vitro biological evaluation against a panel of cancer cell lines, mainly melanoma. As depicted in Scheme 13, both dihydroartemisinin (**2**) and artesunate (**3**) were conjugated with tyrosol **76** in a stereocontrolled manner [68]. Furthermore, depending on the conditions employed, a different regioselectivity in the linkage of tyrosol (**76**) could be achieved. Hybrids **77** and **79** were obtained by conjugation of dihydroartemisinin (**2**) and artesunate (**3**), respectively, with the primary alcohol of tyrosol **76**. On the other hand, the conjugation of dihydroartemisinin (**2**) and artesunate (**3**) with the phenolic moiety of tyrosol **76** led to hybrids **78** and **80**, respectively.

Hybrids **79** and **80** were tested against a panel of cancer cell lines such as HeLa (cervical cancer) and SK-MEL3, SK-MEL24 and RPMI-7951 (metastatic melanoma) as well as normal fibroblast C3PV cells. Both hybrids displayed greater cytoselectivity towards melanoma cancer cell lines with respect to both HeLa and normal fibroblasts. In particular, hybrid **79** showed the best cytotoxicity against metastatic RPMI-7951 melanoma cells with IC_50_ = 0.09 μM, being 12-fold more potent than the parent unconjugated artesunate (**3**) (IC_50_ = 1.08 μM) (Scheme 13). Moreover, hybrid **79** was found to be much less toxic in normal fibroblast C3PV cells (C3PV/RPMI-7951 = 1467) [67]. Interestingly, hybrid **79** showed higher cytotoxic activity compared to hybrid **80** in all cell lines tested, indicating that the regioselective linkage of the alcohol moiety of tyrosol **76** can somewhat influence the hybrid cytotoxicity.

On the contrary, dihydroartemisinin-derived hybrids **77** and **78** showed similar cytotoxicity in the RPMI-7951 melanoma cell line independently of the regioselective linkage of the alcohol moiety of tyrosol **76** (Scheme 13). Hybrids **77** and **78** displayed CC_50_ values towards RPMI-7951 in the same order of magnitude as that of dihydroartemisinin (**2**). However, both hybrids **77** and **78** showed a better safety profile as compared to parent dihydroartemisinin (**2**), being 5.7- and 95-fold less toxic in C3PV normal cells than in RPMI-7951 cancer cells, respectively (Scheme 13) [68].

### 3.5. Artemisinin–Camptothecin Hybrids

**Scheme 14:**
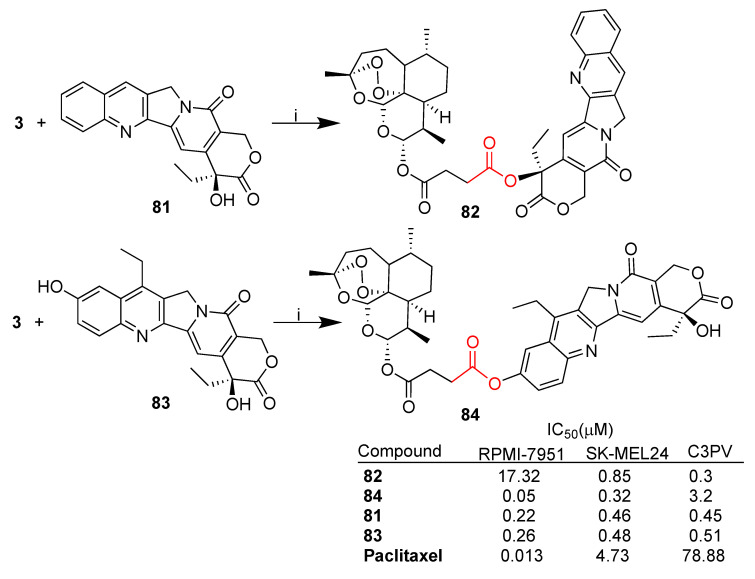
Synthesis of artesunate–camptothecin hybrids and selected biological data. Reaction conditions: (i) EDC, DMAP, DMF, 40–64% yield.

Camptothecin (**81**, Scheme 14) is a natural alkaloid first isolated form the Chinese tree *Camptotheca acuminata.* Camptothecin has been reported to exhibit remarkable anticancer activity against leukemia and several solid tumors including colon and hepatic cancers through inhibition of topoisomerase I [108]. However, camptothecin’s negligible water solubility and poor stability limit its clinical use and have prompted researchers to seek molecular hybridization [109] and prodrug strategies [110].

Recently, Botta et al. reported [69] the synthesis of artesunate–camptothecin hybrids **82** and **84** obtained by the condensation reaction between artesunate (**3**) and camptothecin **81** as well as 7-ethyl-10-hydroxy-camptothecin **83**, the active form of the anticancer drug irinotecan (SN 38) (Scheme 14). Hybrids **82** and **84** were tested in vitro against a selection of melanoma cell lines (RPMI7951 and SK-MEL24) as well as towards normal fibroblast C3PV cells using camptothecins **81** and **83** and paclitaxel as reference drugs [69].

Noteworthy, hybrid **84** showed higher antimelanoma activity towards RPMI-7951 cells than camptothecins and activity similar to that of paclitaxel, as well as a good selectivity index with respect to normal fibroblasts (C3PV/RPMI-7951 = 64).

Both hybrids showed high inhibitory activity against human DNA topoisomerase 1 (hTop1p) in both in vivo and in vitro experiments. In particular, hybrid **84** was active at 3 μM in vivo and at 300 nM in vitro. On the contrary, hybrid **82** was more effective in vivo than in vitro [69].

The overall data indicate the effectiveness of molecular hybridization since hybrid **84** displayed significant enhanced cytotoxicity and cytoselectivity towards cancer cells with respect to normal ones. Moreover, the inhibitory activity of parent camptothecin against hTop1p is preserved in both hybrids **82** and **84**.

### 3.6. Artemisinin–Thymoquinone Hybrids

Thymoquinone is a monoterpene obtained from *Nigella sativa’s* black seed oil [111]. Thymoquinone has received much attention for its wide-ranging pharmacological properties including antioxidant, anti-inflammatory, antidiabetic, antihistaminic, antimicrobial, anticonvulsant and anticancer effects [111]. In the last decade, many studies have been reported on the anticancer effects of thymoquinone on a broad range of cancer types including lung, breast, prostate, gastric, colon, bladder and bone as well as on the use of thymoquinone in combination anticancer therapy [112]. In particular, thymoquinone was reported to exhibit activity against colon cancer by triggering apoptosis through different mechanisms including downregulation of STAT3 activation by reducing JAK2 and c-Src activity, p53- and p21-dependent mechanisms as well as G1-phase cell cycle arrest [112].

**Scheme 15:**
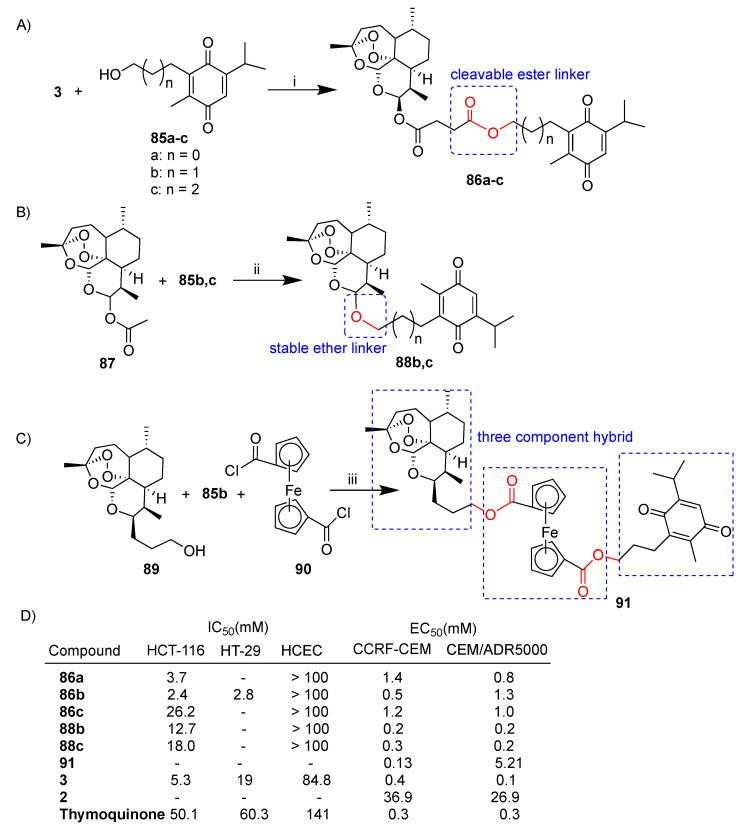
(**A**–**C**): synthesis of artemisinin–thymoquinone hybrids. (**D**): selected biological data. Reaction conditions: (i) DDC, DMAP, DCM, r.t., overnight, 80–86% yield; (ii) TMSOTf, CHCl_3_, 0 °C, 1 h, 32–39% yield; (iii) DCM, DMAP, r.t., overnight, 26% yield.

Tsogoeva’s research group reported extensive studies on the design, synthesis and biological activity of artemisinin–thymoquinone hybrids [70,71,72]. The synthesis of a selection of artemisinin–thymoquinone hybrids is depicted in Scheme 15. Newly synthesized hybrids **86a**–**c**, **88b**,**c** and **91** were evaluated for anticancer activity in HCT-116 and HT-29 colon cancer cell lines [70] and/or sensitive wild-type CCRF-CEM and multidrug-resistant CEM/ADR5000 leukemia cells [71]. Artesunate–thymoquinone hybrids **86a**–**c** were obtained in good yield by the condensation reaction of artesunate (**3**) and thimoquinone derivatives **85a**–**c** (Scheme 15A).

Hybrids **86a**–**c** characterized by labile ester linkers of different lengths were tested on colon cancer and leukemia cells as well as healthy HCEC cells. Notably, the conjugation led in all cases to hybrid compounds being less toxic towards normal cells than parent artesunate (**3**) (Scheme 15D). Among all, hybrid **86b** showed the best anticancer activity against HCT116 cells, being ca. 2- and 21-fold more potent than artesunate (**3**) and thymoquinone, respectively. Similar results were obtained in the case of the HT-29 colon cancer cell line. Also, in the case of leukemia cell lines, hybrid **86b** was the most active of the series with significant cytoselectivity towards sensitive CCRF-CEM. Instead, hybrids **86a**–**c** were found to be around 10-fold less potent than parent artesunate (**3**) in the multidrug-resistant CEM/ADR5000 cell line (Scheme 15D) [71]. A mechanism insight indicated that hybrid **86b** led to an increase in ROS and induced an elevated level of the DNA-damage marker g-H2AX in colon cancer cells [70].

Dihydroartemisinin acetate 87 was reacted with thymoquinone derivatives **85b,c** in order to obtain hybrids **88b,c** characterized by stable ether linkers of different lengths (Scheme 15B) [70]. Both hybrids **88b,c** exhibited similar activity to that of artesunate derivatives 85b,c and thymoquinone towards leukemia cell lines [71] whereas they were found to be 3–4-fold less potent than artesunate (3) alone in the HCT-116 colon cancer cell line (Scheme 15D) [70].

Hybrid 91, a deoxoartemisinin-derived hybrid containing two subunits of ferrocene and thymoquinone prepared as reported in Scheme 15C, was tested against both leukemia CCRF-CEM and CEM/ADR5000 cell lines [72]. Hybrid 91 showed marked cytoselectivity towards sensitive CCRF-CEM with respect to multidrug-resistant CEM/ADR5000 (CCRF-CEM/CEM/ADR5000 = 39) (Scheme 15D) [72]. Most of the artemisinin–thymoquinone hybrids reported showed interesting cytoselectivity which is an important aspect for drug development and one of the main goals of molecular hybridization.

### 3.7. Artemisinin–Chalcone Hybrids

Chalcones are a class of compounds consisting of two aryl rings linked by an α,β-unsaturated ketone moiety and characterized by the common chemical scaffold 1,3-diaryl-2-propen-1-one [113]. Chalcone moieties are common substructures in numerous natural products, including vegetables, fruits, teas, and other plants, belonging to the flavonoid family. Chalcone derivatives are versatile as pharmaceutically active compounds with broad interesting biological activities including anticancer [114], anti-HIV, antimalarial, antioxidant, anti-inflammatory and antiallergic activities [113]. Several chalcone-derived compounds have been approved for clinical use, for instance, metochalcone and sofalcone used as a choleretic drug and antiulcer and mucoprotective drug, respectively [115]. Chalcones have been considered as privileged scaffolds for molecular hybridization to improve biological activities, solubility and/or oral bioavailability as well as to overcome drug resistance. Many chalcone-derived hybrids have been reported in the literature for development of novel anticancer agents with multiple mechanisms of action [116], including artemisinin–chalcone hybrids.

**Scheme 16:**
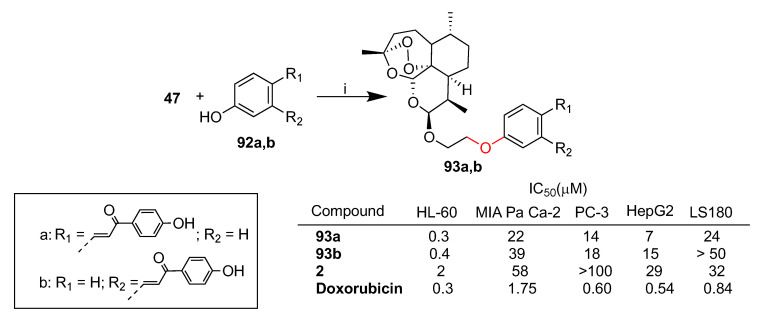
Synthesis of dihydroartemisinin–chalcone hybrids and selected biological data. Reaction conditions: (i) K_2_CO_3_, DMF, DCM, 60 °C, 30 min, 55% yield.

Gau et al. reported a library of dihydroartemisinin–chalcone hybrids conjugated via ether linkers [117]. The hybrids have been evaluated in vitro for their cytotoxicity against a panel of cancer cell lines such as HL-60 (leukemia), MIA PaCa-2 (pancreatic cancer), PC-3 (prostate cancer), HepG2 (hepatocellular carcinoma) and LS-180 (colon cancer) [113]. Hybrids **93a**,**b**, obtained by conjugation of dihydroartemisin derivative **47** and chalcone derivative **92a**,**b**, showed the best activity in HL-60 leukemia cells (Scheme 16). Indeed, hybrids **93a**,**b** (IC_50_ = 0.3 and 0.4 μM, respectively) were found to be 5- and 7-fold more potent than dihydroartemisinin (**2**) (IC_50_ = 2 μM), respectively, and showed comparable cytotoxicity to that of reference drug doxorubicin (IC_50_ = 0.3 μM) (Scheme 16).

A mechanism insight in HL-60 cells was also reported. Both hybrids **93a**,**b** were found to be significantly more effective in inducing apoptotic subG0 population and in triggering mitochondrial membrane potential loss than dihydroartemisinin (**2**).

**Scheme 17:**
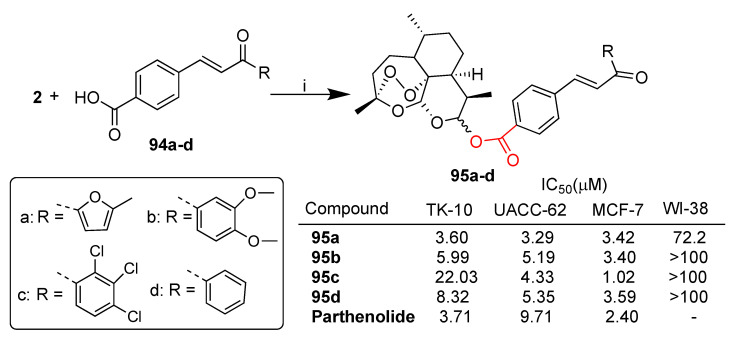
Synthesis of dihydroartemisinin–chalcone hybrids and selected biological data. Reaction conditions: (i) DCI, DCM, r.t., 24 h, 32–61% yield.

Smit et al. reported [75] a library of dihydroartemisinin–chalcone hybrids conjugated via ester linkage that have been evaluated in vitro for their cytotoxicity against TK-10 (renal cancer), UACC-62 (melanoma) and MCF-7 (breast cancer) as well as healthy WI-38 cell lines. The synthesis of selected hybrids **95a**–**d**, prepared by condensation of dihydroartemisinin (**2**) and a series of chalcone derivatives **94a**–**d**, is depicted in Scheme 17. Hybrids **95a**–**c** were found to be highly cytoselective towards cancer cell lines with respect to normal cells. Hybrid **95a** was the most potent of the series against all cancer cell lines tested, showing comparable or higher cytotoxic activity with respect to reference drug parthenolide (Scheme 17).

The structure–activity relationship revealed that the presence of a heterocycle instead of a phenyl ring on the chalcone moiety improved the activity as well as the introduction of electron-withdrawing groups into the phenyl ring.

**Scheme 18:**
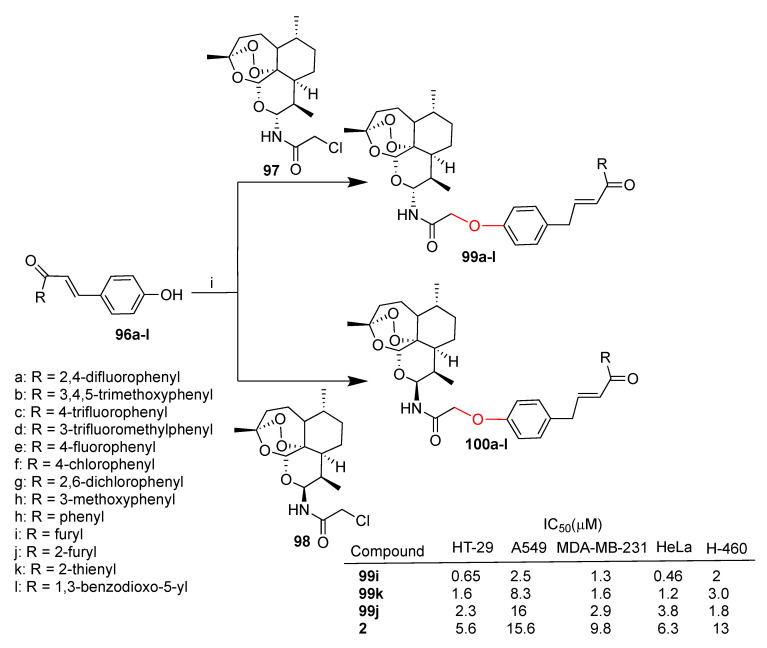
Synthesis of dihydroartemisinin–chalcone hybrids and selected biological data. Reaction conditions: (i) K_2_CO_3_, DMF, NaI, 60 °C, 5 h, 70–90% yield.

Xie et al. reported [73,74] two series of dihydroartemisinin–chalcone hybrids prepared by conjugation through ether linkers of α- and β-C10-aminodihydroartemisinin anomers **97** and **98**, respectively, with chalcone derivatives **96a**–**l** (Scheme 18). Hybrids **99a**–**l** and **100a**–**l** were tested in vitro against the following five cancer cell lines: HT-29 (colon), A549 and A-460 (lung), MDA-MB-231 (breast), HeLa (cervical).

The numerousness of the reported hybrids allowed the establishment of the structure–activity relationship. Particularly, the anomeric configuration at the C-10 position of the dihydroartemisinin moiety does not affect the activity of hybrids. Almost all hybrids of **99a**–**l** were found to be from 2.6- to 40-fold more active than the parent dihydroartemisinin (**2**) in all cancer cell lines tested, proving the effectiveness of the conjugation with a chalcone moiety. Moreover, the presence of a chalcone moiety also improved the cytoselectivity towards HT-29 and HeLa cell lines with respect to MDA-MB-231, A549 and H460 cell lines. By comparing hybrids **99i**–**k** it can be evidenced that the bioisosteric replacement of phenyl (**99i**) with a thiophene (**99k**) and furan (**99j**) rings significantly decreased the activity against the five tumor cell lines tested (Scheme 18).

### 3.8. Artemisinin–Coumarin Hybrids

Coumarin and its derivatives are a family of benzopyrones largely distributed in nature, being present in the seeds, fruits, flowers or roots of various plant species [118,119]. Coumarins are characterized by a two-ring backbone consisting of a phenyl group fused with an α-pyrone ring. Coumarin’s potent biological activity is related to its conjugated electronic system, which is able to react with different molecules including enzymes and receptors of living organisms. Coumarin and its derivatives have been recognized as interesting anticancer candidates due to their ability to induce cell differentiation and apoptosis [118,119,120].

Guo et al. reported the synthesis of several series of dihydroartemisinin–coumarin hybrids via click chemistry [76,77,78] together with some artemisinin–coumarin hybrids via ether linkers [78].

**Scheme 19:**
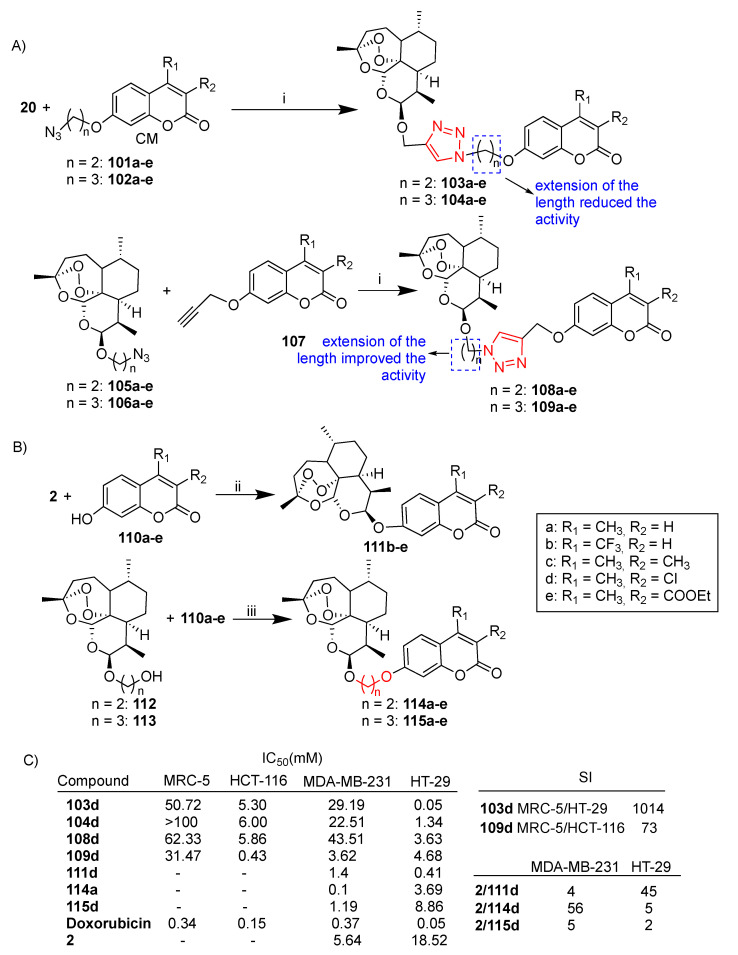
(**A**,**B**) Synthesis of dihydroartemisinin–coumarin hybrids; (**C**) selected biological data. Reaction conditions: (i) CuSO_4_, DMF, sodium ascorbate, r.t., 8–10 h, 20–40% yield; (ii) (CF_3_CO_2_)_2_, Et_3_N, DCM, 0–5 °C, 18 h, 12–17% yield; (iii) K_2_CO_2_, DMF, 60 °C, 5 h, 38–60% yield.

The synthesis of a selection of hybrids such as click hybrids **103a**–**e** and the homologues **104a**–**e** as well as homologue hybrids **108a**–**e** and **109a**–**e** is reported in Scheme 19A.

All hybrids were tested against selected cancer cell lines HCT116 and HT-29 (colon), MDA-MB-231 (breast) and normal MRC-5 fibroblast cells. By comparing hybrids **103a**–**e**, **104a**–**e**, **108a**–**e** and **109a**–**e**, it can be noted that the extension of the length of the alkyl chain between the triazole ring and the coumarin moiety decreased the activity (hybrid activity **103** > **104**) whereas the extension of the length of the alkyl linker between the dihydroartemisinin and triazole moiety increased the activity (hybrid activity **108** < **109**) (Scheme 19C).

The best activity was exhibited by 3-chlorol-4-methyl-coumarin-derived hybrids belonging to the series **d**, suggesting that the different electronic effect of substituents at the C-3 and C-4 skeleton of the coumarin moiety could play a positive role in the cytotoxicity of hybrids. In particular, hybrid **103d** was the most potent with IC_50_ = 0.05 μM, comparable to that of reference drug doxorubicin. Moreover, hybrid **103d** showed the best cytoselectivity towards HT-29 cancer cells with respect to normal MRC-5 cells (MRC-5/HT-29 = 1014) (Scheme 19C). Further studies in HT-29 colon cancer cells showed that hybrid **103d** inhibited proliferation, arrested the cell cycle progression, induced both apoptosis and ferroptosis and inhibited migration [78].

The synthesis of dihydroartemisin-derived fused hybrids **111b**–**e** as well as of hybrids **114a**–**e** and **115a**–**e** conjugated through ether linkers is depicted in Scheme 19B. All hybrids were evaluated in vitro against MDA-MB-231 and HT-29 cancer cells. Among hybrids **111b**–**e**, 3-chloro-4-methyl-coumarin-derived hybrid **111d** showed the best activity towards the HT-29 colon cancer cell line, being 45-fold more potent than unconjugated dihydroartemisinin (**2**) (Scheme 19C). Among hybrids **114a**–**e** and **115a**–**e**, hybrid **114a** showed the best activity towards MDA-MB-231, being 56-fold more active than dihydroartemisinin (**2**) (Scheme 19C) [78].

### 3.9. Artemisinin–Tamoxifen Hybrids

Tamoxifen belongs to a class of anticancer drugs that are selective estrogen receptor modulators extensively used in hormone-dependent breast cancer therapy over recent decades [121,122]. Chronic tamoxifen use can increase the risk of uterine cancer and drug resistance. For these reasons, hybridization of tamoxifen with other anticancer agents may be effective to overcome adverse effects and drug resistance from tamoxifen usage alone.

**Scheme 20:**
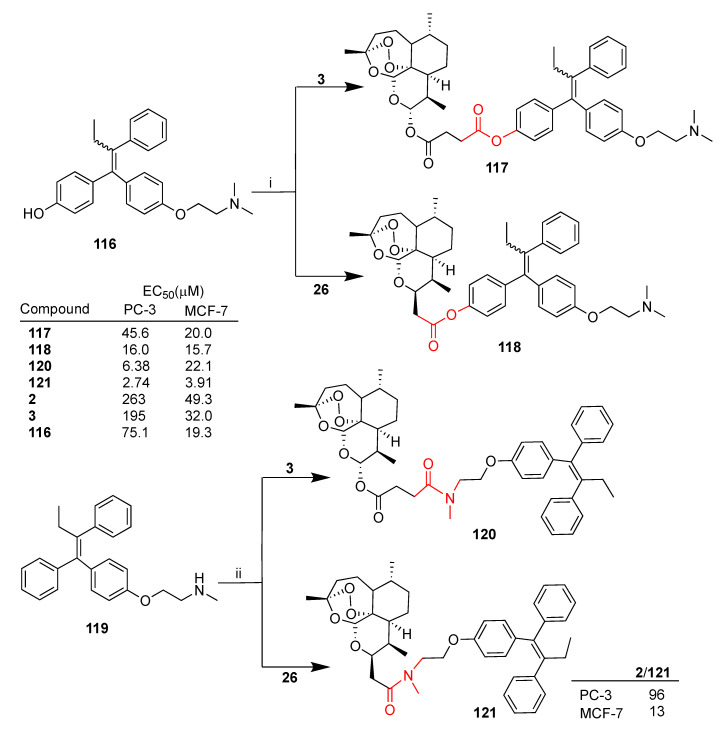
Synthesis of artemisinin–tamoxifen hybrids and selected biological data. Reaction conditions: (i) DMAP, THF, DCC, r.t., 80% yield; (ii) DMAP, THF, EDC, TEA, r.t., 68–75% yield.

Frohlich et al. recently reported [79] the synthesis of some artemisinin–tamoxifen hybrids as well as their biological evaluation against two cancer cell lines, PC-3 (prostate) and MCF-7 (breast) [79]. In particular, both artesunate (**3**) and deoxoartemisinin derivative **26** were reacted with two metabolites of tamoxifen, 2-hydroxytamoxifen **116** (afimoxifene) and (Z)-*N*-desmethyl-tamoxifen **119**, to give the corresponding hybrids **117**, **118** and **120**, **121**, respectively (Scheme 20). Hybrids **117** and **118** presented ester linkers, whereas hybrids **120** and **121** presented amide linkers (Scheme 20). All hybrids displayed higher activity than dihydroartemisinin (**2**) and artesunate (**3**) against both PC-3 and MCF-7. Hybrid **121** was found to be the most potent of the series with EC_50_ = 2.74 and 3.91 μM in PC-3 and MCF-7 cells, respectively. Moreover, hybrid **121** showed marked improved activity compared to dihydroartemisinin (**2**), being 93- and 5-fold more potent in PC-3 and MCF-7, respectively.

### 3.10. Artemisinin–Steroid Hybrids

Steroids are natural organic bioactive compounds present in animals as well as in plants and fungi. Steroids are considered important scaffolds in medicinal chemistry due to their molecular structure as well as to their ability to interact with various biological targets in different pathways [123].

**Scheme 21:**
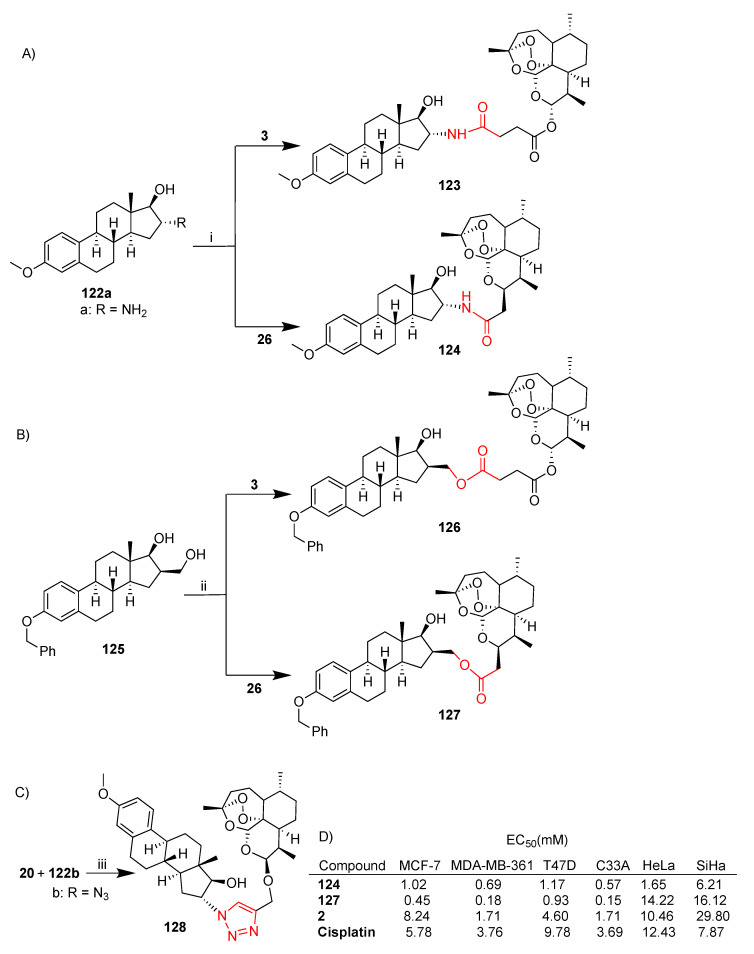
(**A**–**C**): synthesis of artemisinin–estrogen hybrids; (**D**) selected biological data. Reaction conditions: (i) EDC, DCM, ACN, r.t., overnight, 45–80% yield; (ii) DCC, DMAP, DCM, r.t., overnight 55–95% yield; (iii) CuSO_4_, DCM/H_2_O, sodium ascorbate, r.t., overnight, 42% yield.

Frohlich et al. reported [80] a study on the synthesis of a series of artemisinin–estrogen hybrids and their in vitro evaluation against a selection of breast (MCF-7, MDA-MB-231, MDA-MB-361 and T47D) and cervical (HeLa, SiHa and C33A) cancer cell lines [80]. In particular, estradiol amine derivative **122** and estrone derivative **125** were conjugated with both artesunate (**3**) and deoxoartemisinin derivative **26** to give the corresponding hybrids with amide linkers **123**, **124** (Scheme 21A) and with ester linkers **126**, **127** (Scheme 21B). Moreover, estradiol **122** was reacted with dihydroartemisinin alkyne derivative **20** via click reaction to give hybrid **128** characterized by a 1,2,3-triazole linker (Scheme 21C). Almost all hybrids displayed improved growth inhibition than parent artemisinins and estrogen derivatives. In particular, hybrid **127** showed the best activity against breast cancer cell lines with submicromolar EC_50_ values ranging from 0.18 and 0.93 μM and EC_50_ = 0.15 μM in the C33A cervical cancer line with (Scheme 21D). On the other hand, hybrid **124** showed interesting activity towards all cancer cell lines tested (Scheme 21D).

**Scheme 22:**
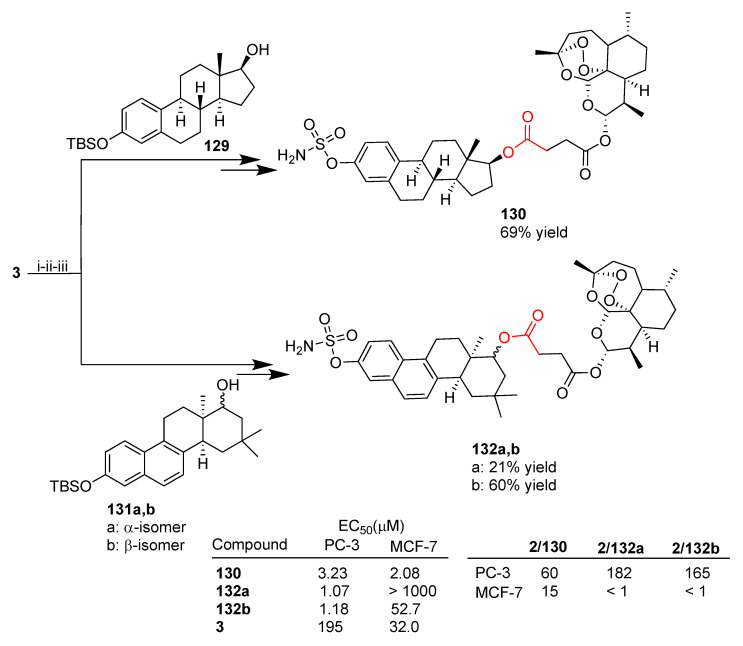
Synthesis of artemisinin–estrogen hybrids and selected biological data. Reaction conditions: (i) EDC, DMAP, THF, r.t.; (ii) TBAF, THF, r.t., 2 h; (iii) NH_2_SO_2_Cl, DMA, r.t., overnight.

More recently, the same research group reported a library of 16 artesunate–estrogen hybrids, prepared via condensation reactions, that were evaluated in vitro on prostate PC-3 and breast MCF-7 cancer cell lines [79]. Among others, estrogen derivatives bearing a sulfamate moiety were used as building blocks. Sulfamate-based estrogen analogues have been reported to display a potential as multitargeting agents for hormone-independent diseases [124]. As reported in Scheme 22, the new hybrids **130** and **132a**,**b** were synthesized through a multistep synthesis involving the condensation reaction between artesunate (**3**) and estrogen derivatives **129** and **131a**,**b** followed by the introduction of the sulfamate moiety and deprotection. All hybrids are characterized by ester linkers. Hybrids **132** were obtained as pure anomers α (**132a**) and β (**132b**) [79].

All hybrids tested were found to be more potent than unconjugated artesunate (**3**) towards PC-3 prostate cancer cells and most of them were found to be more potent towards MCF-7 breast cancer cells as a result of the effectiveness of molecular hybridization. In particular, hybrid **130** was the most active of the whole series towards MCF-7 breast cancer cells, being 15-fold more potent than unconjugated artesunate (**3**). Hybrids **132a**,**b** showed highly marked cytoselectivity towards PC-3 prostate cancer cells with 182- and 165-fold enhanced activity, respectively, compared to parent artesunate (**3**) (Scheme 22).

### 3.11. Artemisinin–Cinnamic Acid Hybrids

Cinnamic acid is a phytochemical isolated from cinnamon bark known for its well-established antimicrobial activity [125]. Cinnamic acid was also reported to have potential anticancer activity in several tumors, therefore it can be considered an interesting scaffold for molecular hybridization [126]

**Scheme 23:**
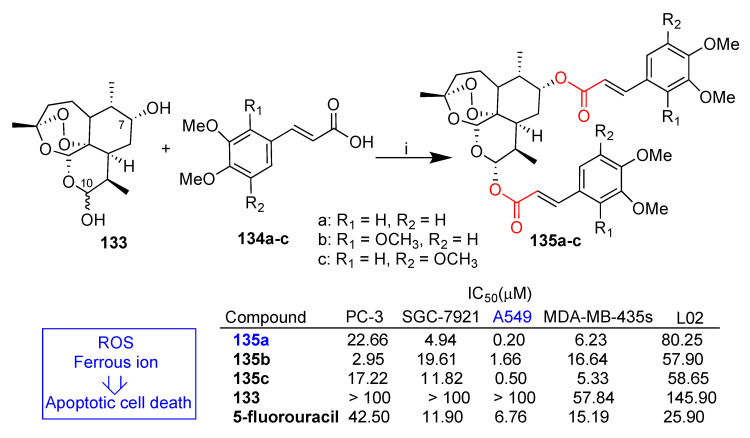
Synthesis of artemisinin–cinnamic acid hybrids and selected biological data. Reaction conditions: (i) EDC, DMAP, DCM, r.t., 12 h, ca. 50% yield.

Xu et al. reported [81] the synthesis of a series of dihydroartemisinin–cinnamic acid hybrids through condensation of a series of cinnamic acids substituted on the benzene ring with dihydroartemisinin derivative **133** (Scheme 23). Disubstituted dihydroartemisinin hybrids prepared by conjugation of suitable cinnamic acid derivatives **134a**–**c** to both C-10 and C-7 hydroxyl groups of dihydroartemisinin derivative **133** have also been reported (Scheme 23) [81]. All hybrids were tested in vitro against four cancer cell lines (PC-3 prostate, SGC-7921 gastric, A549 lung and MDA-MB-435s melanoma) as well as normal L02 cells. Monosubstituted dihydroartemisinin hybrids showed in all cases poor activity whereas the corresponding disubstituted dihydroartemisinin hybrids displayed interesting anticancer activity [81]. In Scheme 23, a selection of disubstituted dihydroartemisinin hybrids **135a**–**c** are reported. In particular, compound **135a** was the most active of the whole series in the A549 cancer cell line with IC_50_ = 0.2 μM and was 34-fold more potent than reference drug 5-fluorouracil and much less cytotoxic in normal L02 cells (L02/A549 = 401). Molecular mechanism studies in the A549 cancer cell line indicated that hybrid **135a** induced apoptosis in a dose-dependent manner. In the same paper [81], it was also reported that both ROS and ferrous ions were involved in the cytotoxicity and apoptosis induced by hybrid **135a**. Therefore, cell lines containing a higher level of ferrous as well as endogenous oxidative stress would be more susceptible to anticancer activity of hybrid **135a**.

### 3.12. Artemisinin–Acridine Hybrids

Acridine derivatives are an important class of heterocycles characterized by a broad spectrum of pharmaceutical properties including anti-inflammatory, antimicrobial, antitubercular, antiparasitic, antimalarial, antiviral as well as anticancer [127]. The variety of pathways involved in acridine anticancer activity, such as inhibition of cell proliferation, apoptosis, cell cycle arrest, retarding migration, invasion and metastasis, encourages acridine’s use as appealing scaffolds for rational design of novel anticancer drug candidates [127,128].

**Scheme 24:**
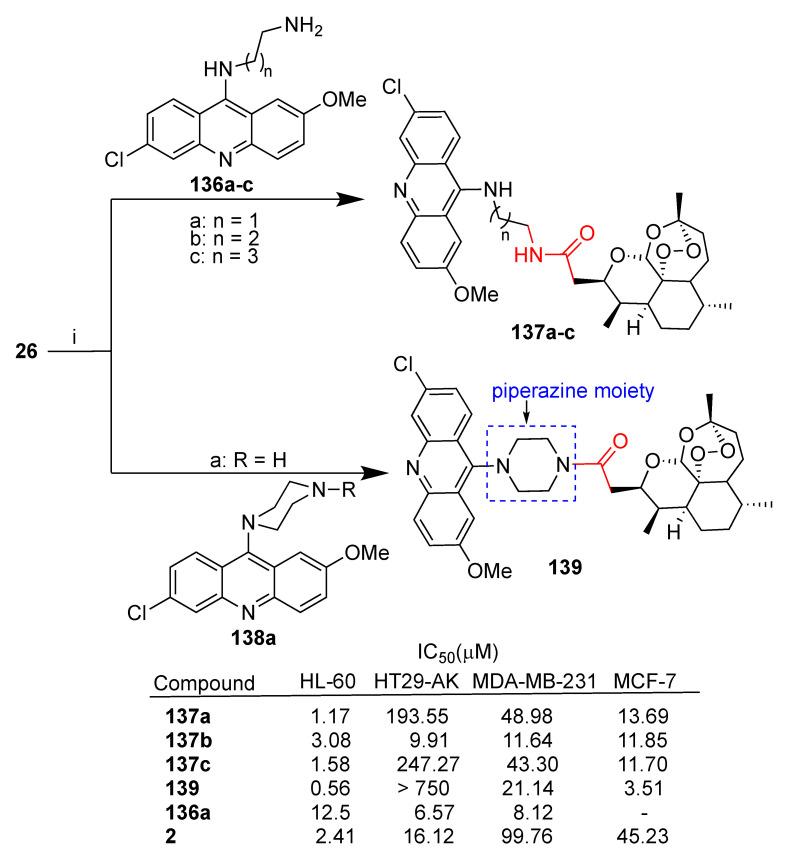
Synthesis of artemisinin–acridine hybrids and selected biological data. Reaction conditions: (i) (COCl)_2_, DCM, r.t., 1.30 h, then NEt_3_, DCM, r.t., 16 h, 55–74% yield.

Jones et al. reported [82] the synthesis of several hybrids obtained by reaction between deoxoartemisin **26** and the appropriate diaminoacridine **136a**–**c** and **138**. The resulting hybrids **137a**–**c**, characterized by amide linkers of different lengths, and hybrid **139**, with a piperazine ring linker, were tested in vitro against a panel of cancer cell lines (leukemia HL-60, colon cancer HT29-AK, breast MDA-MB-231 and MCF-7) in comparison to parent dihydroartemisinin (**2**) and acridine **136a** (Scheme 24). All hybrids tested were found to be cytoselective towards leukemia and breast cancer cell lines, displaying significant enhanced activity with respect to parent dihydroartemisinin (**2**). Compound **137b** is the only hybrid with significant activity towards the HT29-AK colon cancer cell line with 1.6-fold enhanced activity with respect to unconjugated dihydroartemisinin (**2**). This result may be related to the length of the linker that seems to play a role in the cytoselectivity and activity in the series of hybrids **136a**–**c**. Hybrid **136b** was further investigated in HL-60 leukemia cells. Flow cytometry analysis demonstrated that this hybrid can promote apoptotic cell death and bind covalently to intraparasitic cellular targets in the presence of iron (II) [82].

On the other hand, hybrid **139** with the piperazine moiety was the most potent against both HL-60 and MCF-7 cell lines with improved activity compared to parent dihydroartemisinin (**2**) of 6- and 13-fold, respectively (Scheme 24).

**Scheme 25:**
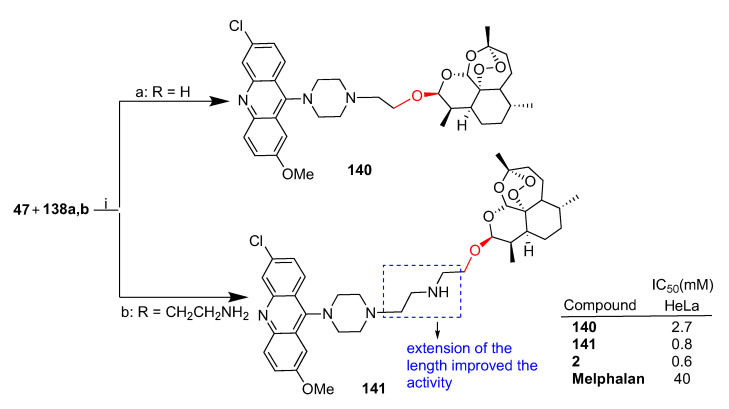
Synthesis of artemisinin–acridine hybrids and selected biological data. Reaction conditions: (i) K_2_CO_3_, KI, ACN, microwave, 10 min, 45 °C, 64–71% yield.

Joubert et al. reported [83] the synthesis of dihydroartemisinin–acridine hybrids obtained by reaction of dihydroartemisinin bromo derivative **47** and a series of diaminoacridine derivatives that were tested against HeLa cervical cancer cells. The synthesis of two hybrids containing a piperazine linker is reported in Scheme 25. The hybrids containing the piperazine ring showed higher activity [83]. In particular, hybrid **141** was the most potent of the whole series with comparable activity to that of parent dihydroartemisinin (**2**). Of note, hybrid **141** with a longer linker displayed 3-fold higher activity than **140**, underlining a possible role of the linker length in the bioactivity.

### 3.13. Artemisinin–Isatin Hybrids

**Scheme 26:**
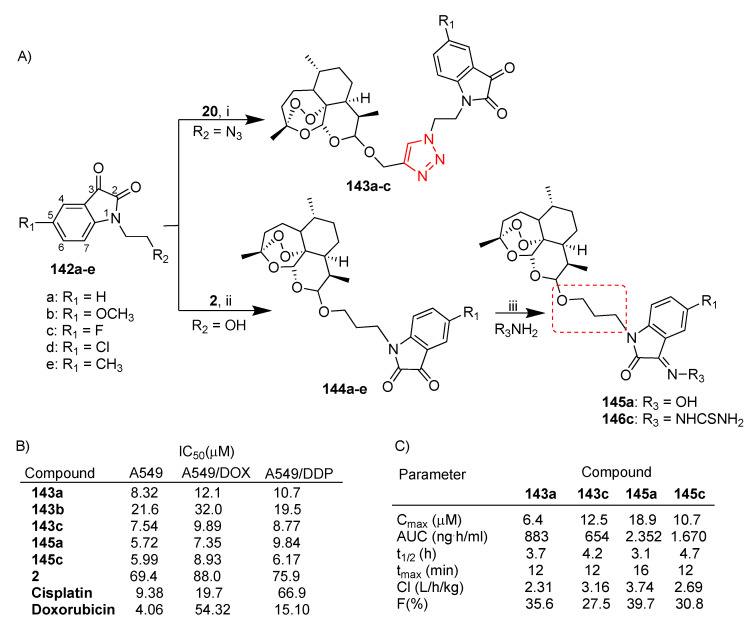
(**A**) Synthesis of dihydroartemisinin–isatin hybrids; (**B**) selected biological data; (**C**) pharmacokinetic data: maximum plasma concentration (C_max_), area under curve (AUC), half-life (t_1/2_), peak time (t_max_), clearance rate (Cl), bioavailability (F). Reaction conditions: (i) CuSO_4_, DMF, 60 °C, 8 h, 28–37% yield; (ii) BF_3_^.^Et_2_O, DCM, 0 °C, overnight, 39–61% yield; (iii) NaHCO_3_, THF, 60 °C, 12 h, 87 and 26% yield.

Isatin, an indole derivative, is a natural alkaloid present in plants of the Isatis genus spread all over the world [129]. Isatin is a synthetic versatile pharmacophore since nitrogen N-1, C-2 and C-3 carbonyl groups as well as any carbon in the benzene ring (Scheme 26) can be modified, leading to a broad spectrum of derivatives with biological properties including, among others, antiviral [130], antimicrobial [131], antibacterial [132] and anticancer [129,133]. Some isatin derivatives have been tested in clinical trials [134] or approved as anticancer drugs [135,136]. Recently, isatin has also attracted attention as a conjugation partner of dihydroartemisin.

Gao et al. reported the synthesis of a large number of dihydroartemisinin–isatin hybrids via both click chemistry [84] and condensation reactions [85] (Scheme 26A). All hybrids were evaluated in vitro for antiproliferative activity in A549 and A549 doxorubicin- and cisplatin-resistant (namely A549/DOX and A549/DDP, respectively) lung cancer cell lines [84,85]. As shown in Scheme 26A, hybrids **143a**–**c**, characterized by the triazole linker, presented different substituents on the C-5 position of the isatin moiety. The SAR indicated that the presence of an electron-withdrawing group such as fluorine (hybrid **143c**) improved the activity whereas the electron-donating group methoxy (hybrid **143b**) led to a significant decrease in the activity (Scheme 26B). Further substitutions of the carbonyl group at the C-3 position of isatin with a hydroxime, alkoxime, benzyloxime and thiosemicarbazide moiety reduced the activity (Scheme 26) [84]. Click hybrids **143a**–**c** were found to be more active (IC_50_ = 7.54–32.0 μM) than unconjugated dihydroartemisinin (**2**) (IC_50_ = 69.4–88.0 μM) in the drug-sensitive and multidrug-resistant A549 cancer cell lines tested (Scheme 26B). On the other hand, all hybrids displayed no cytotoxicity in normal fibroblast HIH/3T3 cells (IC_50_ > 100 μM) [84]. It is worth noting that hybrids **143a**–**c** were found to be at least 6-fold more active than cisplatin in A549/DDP cells (Scheme 26B).

Hybrids **144a**–**e** (Scheme 26A), characterized by a three-carbon alkyl spacer, did not show any activity against all A549 cell lines tested; only hybrid **144c** displayed antiproliferative activity comparable to that of unconjugated dihydroartemisinin (2) (Scheme 26B). On the other hand, the introduction of a hydroxime and a thiosemicarbazide at the C-3 position of the isatin moiety of hybrids **144a**,**c** led to the corresponding hybrids **145a** and **146c** with significant enhanced activity with respect to parent dihydroartemisinin (**2**) (>10-folds) in all cancer cell lines tested (Scheme 26B) [85]. Moreover, hybrids **145a** and **146c** showed interesting activity in both multidrug-resistant 549/DOX and 549/DDP (Scheme 26B) [85].

Hybrids **143a**,**c**, **145a** and **146c** were selected for further pharmacokinetic studies in a CD-1 mouse model by single intravenous administration (dose = 30 mg/kg). The results are reported in Scheme 26C.

**Scheme 27:**
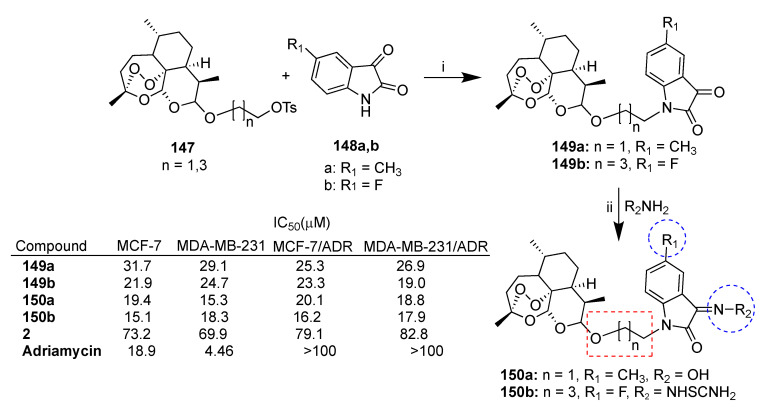
Synthesis of dihydroartemisinin–isatin hybrids and selected biological data. Reaction conditions: (i) K_2_CO_3_, DMF, r.t., overnight; (ii) AcONa, THF/H_2_O, 60 °C, 12 h.

Cao et al. reported [86,87] a series of dihydroartemisinin–isatin hybrids conjugated through a nucleophilic substitution by reacting a series of tosylate derivatives of dihydroartemisinin (**2**) with isatin derivatives substituted at C-3 with different groups such as methoxy, fluorine and methyl. The synthesis of the most potent hybrids of the series is reported in Scheme 27. The new hybrids **149a** [86] and **149b** [87] presented alkyl chains of different lengths and different substituents at the C-5 position of the isatin moiety. Hybrids **149a** and **149b** were in turn modified at the C-3 position of the isatin moiety with, among others, hydroxime and thiosemicarbazide moieties, leading to hybrids **150a** and **150b**, respectively. The new hybrids were tested for their antiproliferative activity against breast cancer cell lines MCF-7 and MDA-MB-231 as well as their corresponding multidrug-resistant cell lines. Most of the hybrids were found to be significantly more potent than parent dihydroartemisinin (**2**) in all cancer cell lines tested and, in particular, hybrids **149a**,**b** and **150a**,**b** were found to be 4- and 5-fold more potent (Scheme 27). Accordingly with previous data reported in the literature [84,85], the nature of the substituents at both C-5 and C-3 positions of isatin influenced the activity, as well as the length of the alkyl chain. It is worth noting that all hybrids were found to be not cytotoxic towards normal MCF-10A breast cells (IC_50_ > 100 μM).

**Scheme 28:**
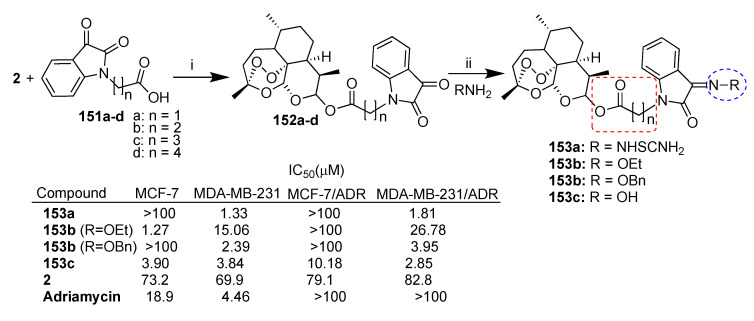
Synthesis of dihydroartemisinin-isatin hybrids and selected biological data. Reaction conditions: (i) HATU, DIEA, DMF, r.t., overnight; (ii) AcONa, THF/H_2_O, 60 °C, 12 h.

Xu et al. reported [88,89] a series of dihydroartemisinin–isatin hybrids conjugated through ester linkers of different lengths. In particular, dihydroartemisinin (**2**) was reacted with isatin carboxylic acid homologue derivatives **151a**–**d** (Scheme 28) and their C-5 derivatives substituted with fluorine, methyl and methoxy moieties. All hybrids were tested for antiproliferative activity against MCF-7 and triple-negative MDA-MB-231 breast cancer cell lines as well as their corresponding multidrug-resistant MCF-7/ADR and MDA-MB-231/ADR. The synthesis of the most potent hybrids of the series **153a**–**c** is reported in Scheme 28. Hybrid **153a** [89], presenting the shortest linker (two-carbon alkyl chain) and the thiosemicarbazide moiety at the C-3 position of isatin, showed high activity and cytoselectivity, being 53- and 46-fold more potent than parent dihydroartemisinin (**2**) towards triple-negative MDA-MB-231 and MDA-MB-231/ADR breast cell lines and 75- and 55-fold more potent than in MCF-7 and MCF-7/ADR cell lines, respectively (Scheme 28). Analogously, hybrid **153b** (R = OBn) [88], with a three-carbon alkyl chain and the benzoxime moiety at the C-3 skeleton of isatin, showed a similar activity profile to that of hybrid **153a** (Scheme 28). On the contrary, hybrid **153b** (R = OEt) [88], with a three-carbon alkyl chain and the ethoxime moiety at the C-3 position of isatin, was found to be cytoselective towards MCF-7 cells with IC_50_ = 1.27 μM (MDA-MB-231/MCF-7 = 11.8) (Scheme 28). Hybrid **153c** [89], with a four-carbon alkyl chain and the oxime moiety at the C-3 position of isatin, showed interesting activity in all breast cancer cell lines tested (IC_50_ = 2.85–10.18 μM). In particular, hybrid **153c** was found to be 29-fold more active than dihydroartemisinin (**2**) in multidrug-resistant triple-negative MDA-MB-231/ADR (Scheme 28). The overall data indicated that the conjugation of dihydroartemisinin (**2**) with isatin through ester bonds could be helpful to overcome drug resistance and to tackle triple-negative breast cancer.

### 3.14. Artemisinin–Sulfasalazine Hybrid

**Scheme 29:**
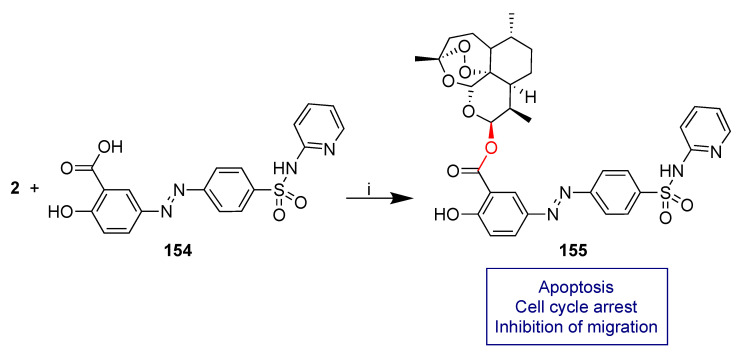
Synthesis of dihydroartemisinin–sulfasalazine hybrid. Reaction conditions: (i) DCC, DMAP, ACN, 40 °C, 8 h 21% yield.

Sulfasalazine (**154**, Scheme 29) is an anti-inflammatory drug in clinical use for chronic diseases such as ulcerative colitis and Crohn’s disease [137] and rheumatoid arthritis [138]. Sulfasalazine’s anticancer activity has also been reported in glioma [139,140] and other cancers [141,142]. Moreover, it has been employed as a chemosensitizer in radiotherapy [140,143] and chemotherapy [144,145,146,147].

Ackermann et al. recently reported a study on the anticancer activity in selected glioma cell lines of dihydroartemisinin–sulfasalazine fused hybrid **155** obtained by condensation reaction of dihydroartemisinin (**2**) and sulfasalazine **154** (Scheme 29) [90].

Hybrid **155** showed significant improved anticancer activity in U87 and TN22 human glioma cells with respect to both unconjugated dihydroartemisinin (**2**) and sulfasalazine **154** as well as their 1:1 combination. In particular, treatment of TN22 cells with 5 μM of hybrid **155** led to a 60% reduction of cell viability whereas unconjugated dihydroartemisinin (**2**) and dihydroartemisinin (**2**) and sulfasalazine **154** 1:1 combinations reduced the cell viability by only 20%. No effect on cell viability was found by treatment with sulfasalazine **154** alone. These data prove a relevant effectiveness of molecular hybridization. A deep mechanism insight demonstrated that the remarkable reduction of cell viability can be attributed to apoptosis and cell cycle arrest. Moreover, hybrid **155** showed significant inhibition of cell migration which is an important property to fight malignancies with invasive growth such as glioma.

## 4. Conclusions

The research on artemisinin’s anticancer activity has demonstrated that despite challenges, repurposing of artemisinin and its derivatives as anticancer drugs is possible. Indeed, artemisinins have shown anticancer activity against different cancer types by acting through various pathways. Anticancer efficacy of artemisinins has also been proved in in vivo studies with evidence of tumor growth inhibition in xenograft models. Human trials reported so far have demonstrated that some artemisinins are safe and have significant anticancer efficacy. However, artemisinins are characterized by poor pharmacokinetic properties such as low tissue distribution and short half-life that limit their applicability in clinical anticancer therapy. To avoid these limitations, different strategies have been implemented, such as the use of combination therapy and nanoformulations, that can overcome the pharmacokinetic barriers of artemisinins, as well as molecular hybridization. This last option can help not only to overcome the intrinsic limitations of artemisinins but also to improve anticancer activity by lowering the toxicity as a result of enhanced cytoselectivity and to improve the efficacy against multidrug-resistant cancers. The discovery of artemisinin’s anticancer activity is relatively recent, therefore, the research on artemisinin moiety molecular hybridization has been developed mainly over the last 10–15 years. Although many efforts have been made, as demonstrated by the numerousness of the hybrids reported in the literature, the lack of systematic studies on the molecular mechanism of action of hybrids as well as the lack of in vivo studies still limit the development of new artemisinin-based hybrid drugs. Indeed, while many hybrids among the herein reported ones showed interesting improved anticancer activity against selected cancer cell lines, few cases have been extensively studied, for instance, ursodeoxycholic bile acid hybrids 13 and 22 in hepatocellular carcinoma [45,46], cinnamic acid hybrid **135a** in lung carcinoma [81] and sulfasalazine hybrid 155 in glioma [90]. Up to now, studies in xenograft models have been restricted to very few cases such as ursodeoxycholic bile acid hybrid 25 in lung cancer [54] and quinazoline hybrid 69 in colon cancer [64]. More in-depth studies into these aspects are required. The eventual development of artemisinin-based hybrids into drugs for cancer chemotherapy can have great relevance.

## Figures and Tables

**Table 1:** Summary of included Artemisinin hybrids.

Hybrid	Hybridization Chemistry	Cancer Cell Type	Ref.	Comments
Artemisinin–bile acid	Ester	Leukemia, B-cell lymphomas and hepatocellular carcinoma	[44,45,53]	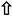 Cytotoxic activity Apoptotic cell death + Safety profile
	Triazole click	Leukemia and hepatocellular carcinoma	[44,46]	
	Ether	Lung, ovarian, prostatic, cervical and renal cell carcinoma	[54]	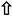 Cytotoxic activity 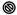 In vivo tumor growth Immunotherapy effects
	Amide	Sensitive and multidrug-resistant leukemia	[55]	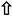 Cytotoxic activity
Artemisinin–quinoline	Amide	Leukemia, oral squamous carcinoma, lung and colon	[56,57,58]	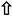 Cytotoxic activity + Safety profile
	Hydrazone	Breast	[59,60]	More potent than reference drugs Apoptotic cell death
	Diamine/piperazine	Renal, melanoma and breast	[61,62]	More potent than reference drugs
Artemisinin–quinazoline	Amide	Sensitive and multidrug-resistant leukemia	[63]	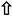 Cytotoxic activity
	Ether/ester/chiral amide	Colon, melanoma	[64]	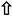 Cytotoxic activity 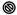 In vivo tumor growth
Artemisinin–nitrogen mustard	Ester	Leukemia	[65]	More potent than reference drugs Apoptotic cell death
	Amide	Ovarian	[66]	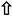 Cytotoxic activity More potent than reference drugs Apoptotic cell death Migration inhibition
Artemisinin–tyrosol	Ester	Cervical, melanoma	[67,68]	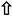 Cytotoxic activity + Safety profile
Artemisinin–camptothecin	Ester	Melanoma	[69]	More potent than reference drugs
Artemisinin–thymoquinone	Ester/ether	Colon, sensitive and multidrug-resistant leukemia	[70,71]	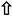 Cytotoxic activity + Safety profile
	Ferrocene–ester	Sensitive and multidrug-resistant leukemia	[72]	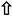 Cytotoxic activity
Artemisinin–chalcone	Ether	Leukemia, pancreatic, prostate, hepatocellular carcinoma, colon, lung, breast and cervical	[69,73,74]	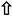 Cytotoxic activity Apoptotic cell death
	Ester	Renal, melanoma and breast	[75]	+ Safety profile
Artemisinin–coumarin	Triazole click/ether	Colon and breast	[76,77,78]	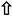 Cytotoxic activity Apoptotic and ferroptotic cell death Migration inhibition + Safety profile
Artemisinin–tamoxifen	Ester/amide	Prostate and breast	[79]	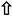 Cytotoxic activity
Artemisinin–steroid	Ester	Prostate, breast and cervical	[79,80]	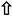 Cytotoxic activity More potent than reference drugs
	Amide/triazole click	Breast and cervical	[80]	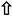 Cytotoxic activity More potent than reference drugs
Artemisinin–cinnamic acid	Ester	Prostate, gastric, lung and melanoma	[81]	More potent than reference drugs + Safety profile Apoptotic cell death
Artemisinin–acridine	Amide	Leukemia, colon and breast	[82]	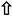 Cytotoxic activity Apoptotic cell death
	Ether	Cervical	[83]	More potent than reference drugs
Artemisinin–isatin	Triazole click	Lung	[84]	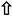 Cytotoxic activity More potent than reference drugs
	Ether	Lung, breast	[85,86,87]	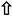 Cytotoxic activity + Safety profile
	Ester	Breast	[88,89]	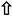 Cytotoxic activity
Artemisinin–sulfasalazine	Ester	Glioma	[90]	Apoptotic cell death Migration inhibition

The 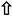 symbol marks the cytotoxic effect of hybrids with respect to artemisinins. The 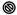 symbol marks the tumor suppressor effect. The + symbol marks the safety profile of hybrids in healthy cells.

## Data Availability

Not applicable.
